# Anti‐Oxidative and Anti‐Inflammatory Micelles: Break the Dry Eye Vicious Cycle

**DOI:** 10.1002/advs.202200435

**Published:** 2022-04-18

**Authors:** Su Li, Zhouyu Lu, Yue Huang, Yin Wang, Qiao Jin, Xingchao Shentu, Juan Ye, Jian Ji, Ke Yao, Haijie Han

**Affiliations:** ^1^ Eye Center The Second Affiliated Hospital School of Medicine Zhejiang University 88 Jiefang Road Hangzhou 310009 P. R. China; ^2^ MOE Key Laboratory of Macromolecule Synthesis and Functionalization of Ministry of Education Department of Polymer Science and Engineering Zhejiang University Hangzhou 310027 P. R. China; ^3^ School of Pharmacy Shanghai Jiao Tong University 800 Dongchuan Road Shanghai 200240 P. R. China; ^4^ Zhejiang Provincial Key Lab of Ophthalmology Zhejiang University 88 Jiefang Road Hangzhou 310009 P. R. China

**Keywords:** anti‐inflammation, anti‐oxidant stress, dry eye, micelles, vicious cycle

## Abstract

Dry eye disease (DED) impacts ≈30% of the world's population and causes serious ocular discomfort and even visual impairment. Inflammation is one core cause of the DED vicious cycle, a multifactorial deterioration in DED process. However, there are also reactive oxygen species (ROS) regulating inflammation and other points in the cycle from the upstream, leading to treatment failure of current therapies merely targeting inflammation. Accordingly, the authors develop micelle‐based eye drops (more specifically p38 mitogen‐activated protein kinases (MAPK) inhibitor Losmapimod (Los)‐loaded and ROS scavenger Tempo (Tem)‐conjugated cationic polypeptide micelles, designated as MTem/Los) for safe and efficient DED management. Cationic MTem/Los improve ocular retention of conjugated water‐soluble Tem and loaded water‐insoluble Los via electrostatic interaction with negatively charged mucin on the cornea, enabling an increase in therapeutic efficiency and a decrease in dosing frequency. Mechanistically, MTem/Los effectively decrease ROS over‐production, reduce the expression of proinflammatory cytokines and chemokines, restrain macrophage proinflammatory phenotypic transformation, and inhibit cell apoptosis. Therapeutically, the dual‐functional MTem/Los suppress the inflammatory response, reverse corneal epithelial defect, save goblet cell dysfunction, and recover tear secretion, thus breaking the vicious cycle and alleviating the DED. Moreover, MTem/Los exhibit excellent biocompatibility and tolerability for potential application as a simple and rapid treatment of oxidative stress‐ and inflammation‐induced disorders, including DED.

## Graphical abstract

MTem/Los with anti‐oxidative and anti‐inflammatory functions are reported to effectively reduce overproduced reactive oxygen species and secretory proinflammatory cytokines and chemokines, restrain macrophage proinflammatory phenotypic transformation, and inhibit cell apoptosis, which results in reversing corneal epithelial defect, saving goblet cell dysfunction, and recovering tear secretion, ultimately breaking the vicious cycle and alleviating dry eye disease.

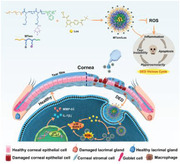

## 1 Introduction

Dry eye disease (DED), a multifactorial disorder primarily originating from hyperosmolarity of tear film, can result in ocular discomfort and even visual impairment, seriously affecting patients’ life quality.^[^ ^1^, ^2^ ^]^ Currently, DED affects ≈30% of the world's population and has become the most common ocular surface disease worldwide, posing an enormous economic burden to society.^[^ ^3^, ^4^ ^]^ Although the pathogenesis of DED remains only partially elucidated, inflammation, as the consequence of both earlier innate immune response and following adaptive response, has been identified as a critical contributor that can initiate dry eye's vicious cycle. The vicious cycle refers to a malignant development interacted among inflammation, hyperosmolarity, cell apoptosis, and tear film decline that deteriorates dry eye syndrome.^[^ ^5^ ^]^ This multi‐factor‐formed cycle severely hinders effective DED management using any currently available treatment. Corticosteroids, the most popular anti‐inflammatory options, yet are largely limited for long‐term application due to severe risks such as glaucoma and cataract. Three immune‐modulatory agents, including cyclosporine‐A eye drops (Restasis), have been approved by Food and Drug Administration (FDA) for DED treatment. Unfortunately, such agents require from weeks to months to take effect and are accompanied by the defect of innate inflammatory mediator suppression.^[^ ^6^ ^]^ Furthermore, these treatments generally require frequent administration with high drug concentration due to the rapid elimination of the drug on the ocular surface, which may potentially generate adverse effects such as ocular burning as well as visual acuity and result in poor patient compliance.^[^ ^7^ ^]^ Therefore, there is an imperative need to develop safe and effective DED therapeutic strategies.

The activation of mitogen‐activated protein kinases (MAPK) and nuclear factor‐*κ*B (NF‐*κ*B) by direct hyperosmotic stress (HS) in corneal epithelial cells are core components of the innate immune system as well as the primary stage in the increscent process of DED.^[^ ^8^ ^]^ MAPKs are one of the most significant conserved protein kinases families in mammals that link extracellular signals to the intracellular machinery to regulate a plethora of cellular processes such as inflammation, immune response, and apoptosis. p65 is a core member of the NF‐*κ*B family which plays key roles in inflammation activation^[^ ^9^, ^10^ ^]^ and may act downstream of the p38 MAPK pathway^[^ ^11^ ^]^ and consequently promote the expressions of many genes, including matrix metalloproteinases (MMPs), for example, MMP‐9 and proinflammatory cytokines such as Interleukin 1β (IL‐1*β)*, causing corneal barrier dysfunction. The excessive IL‐1*β* release also amplifies the inflammatory cascades and maturates antigen‐presenting cells (APCs) (e.g., macrophages). As a result, the activated APCs bring about an adaptive immune response with long‐term persistence, making it more prone to flare up inflammation in DED. Apart from presenting antigens, activated APCs also secrete proinflammatory cytokines, causing epithelial cell damage.^[^ ^12^, ^13^ ^]^ Together, these diverse responses promote cell apoptosis, a significant pathologic discovery in DED. Conclusively, to break DED's vicious circle in the early stages, inflammation alleviation by inhibiting the MAPK signaling pathway in both epithelium and resident immune cells is a promising strategy.

p38 MAPK is receiving increasing attention as a significant target in anti‐inflammatory treatment,^[^ ^14^, ^15^ ^]^ and the suppression of inflammation dominated by p38 MAPK could be a distinguished alternative, enabling inhibition of inflammatory cytokine expression and clearance of apoptotic cells. However, attempts to merely use small molecular inhibitors of p38 MAPK to treat multifactorial and chronic diseases such as DED might be compromised in therapeutic efficacy. This is because, beyond the DED vicious cycle, there is also an upstream mediator due to excess production of reactive oxygen species (ROS) characterized as oxidative stress, which plays a central role in DED development.^[^ ^16^ ^]^ Notably, excessive ROS not only regulates p38 MAPK and downstream cytokine transcription as an upstream activator,^[^ ^17^ ^]^ it also directly causes damage to cellular DNA, protein, lipid, and even collateral tissues.^[^ ^18^, ^19^ ^]^ The strategy that “cut off the head of the snake”, namely scavenging overproduced ROS by antioxidants, is thus developed for alleviating oxidative stress‐driven DED.^[^ ^3^, ^20^, ^21^, ^22^ ^]^ The superoxide dismutase mimetic compound Tempo (Tem) is a cheap and stable nitroxide radical but has robust antioxidant activity, and its derivatives, nanomedicines, and gels have been widely applied in various oxidative stress injuries such as hypertension,^[^ ^23^ ^]^ atherosclerosis,^[^ ^24^ ^]^ inflammatory bowel disease,^[^ ^25^ ^]^ and myocardial infarction.^[^ ^26^ ^]^ However, since the small, hydrophilic Tem can be rapidly (15–30 s after application) cleared away after topical administration, its ocular application is critically limited.^[^ ^27^ ^]^ Moreover, efficiently accommodating Tem into hydrophobic micellar nanoparticles or polymer microparticles is full of challenges.^[^ ^28^ ^]^

**Figure 1:**
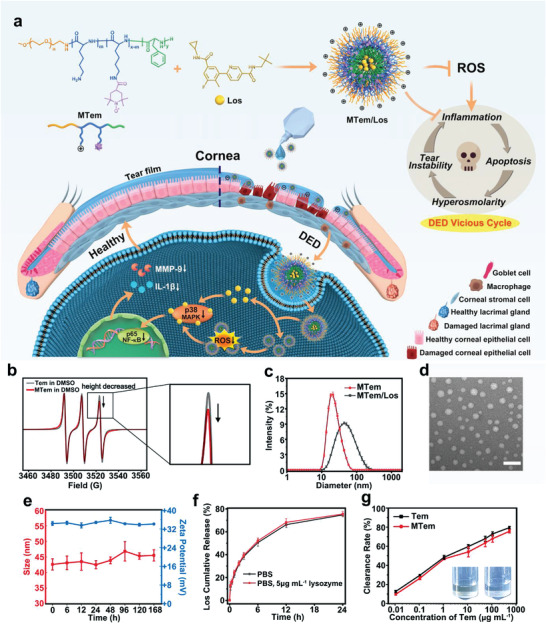
Illustrative scheme and characterization of the synthesized polypeptides, MTem, and MTem/Los. a) Illustration of engineering anti‐oxidative and anti‐inflammatory MTem/Los with prolonged ocular surface retention time and its therapeutic effect and mechanism via suppressing ROS‐mediated p38 MAPK pathway and breaking the vicious cycle in DED. b) X‐band EPR spectra of Tem and MTem in DMSO. c) The hydrodynamic diameter of MTem and MTem/Los. d) TEM image of MTem/Los. The scale bar is 200 nm. e) Changes of hydrodynamic diameter and zeta potential of MTem/Los over different times measured by DLS. f) In vitro drug release profile of Los encapsulated MTem/Los micelles with/without 5 µg mL^−1^ lysozyme in PBS. g) Antioxidant capacity of Tem and MTem by measuring the hydrolysis degree of H_2_O_2_ through a hydrogen peroxide assay kit. Inset pictures: Color change of PEG‐*b*‐(Lys‐*graft*‐Tem‐*co*‐Phe) before (left) and after (right) the addition of ascorbic acid. Data were presented as mean ± SD; *n* = 3.

Losmapimod (Los) is a safe and well‐tolerated p38 MAPK inhibitor that shows remarkable inhibiting effects in cardiological, pulmonary, and neural clinical experiments.^[^ ^29^, ^30^, ^31^ ^]^ Based on the potency of Tem and Los for resolution of inflammation and our existing expertise in nanocarriers for ocular surface drug delivery,^[^ ^32^, ^33^, ^34^ ^]^ we developed a novel, safe, and highly effective micellar eye drops that mainly consist of the Los‐loaded and Tem‐conjugated cationic polypeptide micelles, MTem/Los, for the superior combat against DED vicious cycle (**Figure** **1**a). The MTem/Los micelles were prepared simply by the self‐assembly of cationic Tem‐conjugated polypeptides with the Los package. The mucoadhesive nature of MTem/Los, originating from the positive charge of unreacted lysine segments, results in improved contact with the ocular surface, while its small size allows better tissue penetration, ultimately improving in vivo bioavailability of conjugated Tem and Los delivery.^[^ ^35^ ^]^ In this article, we proved that mechanically, MTem/Los reduced the production of ROS, pro‐inflammatory and immune chemokines, and it also inhibited macrophage pro‐inflammatory phenotypic transformation as well as epithelial cell apoptosis. Therapeutically, the dual‐functional MTem/Los could efficiently suppress the inflammatory response, reverse corneal epithelial defect, and recover tear secretion as well as damages on the conjunctiva and lacrimal gland, thus alleviating the DED. Our MTem/Los exhibited outstanding biosafety in vitro and in vivo and provided a novel insight for the development of convenient, efficient, and combined treatment for DED and other oxidative stress‐ and inflammation‐related diseases.

## 2 Results

### 2.1 Preparation and Characterization of Anti‐Oxidative and Anti‐Inflammatory Micelles

The synthesis of the cationic amphiphilic anti‐oxidative polypeptide (PEG‐*b*‐(Lys‐*graft*‐Tem‐*co*‐Phe) was conducted according to the synthetic route shown in Figure S1, Supporting Information.

Polypeptide precursors protected with carbobenzyloxy (CBz) groups (PEG‐*b*‐(Lys(CBz)‐*co*‐Phe) were first synthesized by N‐Carboxyanhydride (NCA) ring‐opening copolymerization of Lys(CBz)‐NCA and Phe‐NCA monomers using methoxypolyethylene glycol amine (mPEG‐NH_2_) as the initiator, followed by de‐protection of CBz group using trifluoroacetic acid and hydrobromic acid to obtain PEG‐*b*‐(Lys‐*co*‐Phe), where Lys and Phe acted as the hydrophilic (cationic) segment and the hydrophobic segment, respectively. The carboxyl groups in the Tem were then covalently linked to the amino groups of the synthesized polypeptide side chains by amide reaction to obtain PEG‐*b*‐(Lys‐*graft*‐Tem‐*co*‐Phe).

The chemical structures of as‐synthesized polypeptides, including PEG‐*b*‐(Lys(CBz)‐*co*‐Phe), PEG‐*b*‐(Lys‐*co*‐Phe), and PEG‐*b*‐(Lys‐*graft*‐Tem‐*co*‐Phe), were characterized by proton nuclear magnetic resonance (^1^H‐NMR) as shown in Figure S2–S4, Supporting Information. By comparing the well‐defined peak integrals of the methoxy group in mPEG‐NH_2_ with Lys(CBz)‐NCA and Phe‐NCA, the polymerization degrees of Lys(CBz)‐NCA and Phe‐NCA were calculated to be 26 and 19, respectively (Figure S2, Supporting Information). These results verified the successful synthesis of PEG‐*b*‐(Lys(CBz)‐*co*‐Phe). After de‐protection of CBz groups in PEG‐*b*‐(Lys(CBz)‐*co*‐Phe) treated by hydrobromic acid solution (33 wt% in acetic acid), the characteristic peaks of CBz groups at *δ* 5.0 and 7.3 ppm disappeared as shown in Figure S3, Supporting Information. PEG‐*b*‐(Lys‐*co*‐Phe) was successfully prepared with complete removal of CBz groups and its molecular weight was calculated as 8120. Because of the existence of single unpaired electrons in Tem, it is difficult to acquire the ^1^H‐NMR spectrum of PEG‐*b*‐(Lys‐*graft*‐Tem‐*co*‐Phe) to conduct structural elucidation without adding reducing agents. Accordingly, diphenylhydrazine was added in situ in the NMR tube for quenching the radical to form hydroxylamine amenable to ^1^H‐NMR spectroscopy.^[^ ^26^, ^36^ ^]^ Specifically, the appearance of characteristic peaks of methyl groups in Tem at *δ* 1.1 ppm confirmed the successful synthesis of PEG‐*b*‐(Lys‐*graft*‐Tem‐*co*‐Phe) as shown in Figure S4, Supporting Information. The position of these Tem peaks is consistent with that of methyl groups in Tem at *δ* 0.8‐1.2 ppm according to the previously reported literature.^[^ ^26^, ^36^, ^37^ ^]^ In addition, UV–vis spectroscopy demonstrated successful conjugation of Tem to the polypeptide (Figure S5, Supporting Information).

Electron paramagnetic resonance (EPR) measurement was employed to further determine the grafting degree of Tem radicals in PEG‐*b*‐(Lys‐*graft*‐Tem‐*co*‐Phe) (Figure S6, Supporting Information, and Figure 1b). Free nitroxide radicals typically exhibited a sharp triplet EPR spectrum due to the hyperfine interaction of the free electron with the natural isotope of nitrogen ^14^N (*I* = 1). The EPR spectrum of Tem in DMSO solution indicated three equally intense peaks (Figure 1b). The EPR spectrum of PEG‐*b*‐(Lys‐*graft*‐Tem‐*co*‐Phe) dissolved in DMSO featured three peaks appearing at the same magnetic field strengths as free Tem. However, the height of the high field peak at 3522 G decreased compared to the peaks at 3491 and 3506 G, which is ascribed to the hindrance in the movement of covalently conjugated Tem molecules.^[^ ^26^ ^]^ Calculation based on EPR tests revealed approximately 6 Tem units in each polypeptide based on a free Tem radical calibration curve (Figure S6, Supporting Information and Figure 1b).^[^ ^38^ ^]^ It is worth noting that, as the inset pictures in Figure 1g shows, PEG‐*b*‐(Lys‐*graft*‐Tem‐*co*‐Phe) in phosphate‐buffered saline (PBS, pH 7.4) was orange as a result of the color of nitroxide radicals from Tem (left). This orange color could be bleached by reduction with ascorbic acid (right). Together, these results substantiate the successful preparation of PEG‐*b*‐(Lys‐*graft*‐Tem‐*co*‐Phe) with the expected structure.

The Los‐loaded anti‐oxidative micelles, MTem/Los, were prepared by the conventional dialysis method, and their self‐assembly behaviors were evaluated by dynamic light scattering (DLS) and transmission electron microscope (TEM) analysis (Figure 1c,d). DLS results showed that the average size of MTem increased from 26.7 to 42.6 nm, with PDI from 0.205 to 0.253 after loading Los. The TEM images of the self‐assembled MTem/Los confirmed the presence of uniformly dispersed spherical morphologies. Meanwhile, MTem/Los exhibited a positive surface charge, with a zeta potential of +34.3 mV (Figure 1e), originating from their unreacted and exposed amino groups. These positively charged micelles were expected to have a high binding affinity to anionic mucosal sialic acid residues in the corneal and conjunctival surfaces due to the electrostatic interaction, which was beneficial for enhancing the ocular retention time.^[^ ^33^, ^39^ ^]^ Moreover, the size and zeta potential of MTem/Los kept constant with negligible changes in PBS for one week, possessing robust structural stability (Figure 1e). The drug loading efficiency (DLE) of Los in the MTem/Los was 36.1%, with drug loading content (DLC) 1.8% determined by High‐Performance Liquid Chromatography (HPLC) compared to the calibration curve of free Los (Figure S7, Supporting Information). Of particular note is that MTem/Los could reach a much higher DLE and DLC. But we adopted a relatively low DLC of Los in MTem/Los for balancing the anti‐oxidative from MTem and anti‐inflammatory from Los. Since lysozyme is the most prevalent protein in tears and plays an important role in the innate immune system, the in vitro drug release behavior of MTem/Los with/without 5 µg mL^−1^ lysozyme was subsequently investigated.^[^ ^40^ ^]^ As shown in Figure 1f, no matter whether adding 5 µg mL^−1^ lysozyme, the drug release behavior of MTem/Los almost kept the same, with approximately 23% and 51% of loaded Los released from MTem/Los in 1 and 6 h, respectively. Meanwhile, the hydrodynamic diameter of MTem/Los kept almost unchanged after adding lysozyme (Figure S8, Supporting Information), indicating that lysozyme has little influence on drug release behavior and size stability of MTem/Los.

Since Tem is a superior stable radical and ROS trapper, we studied whether the micelles derived from self‐assemblies of Tem integrated polypeptide maintained the parent Tem's ability to scavenge ROS. To avoid the possible interference of released Los, anti‐oxidative micelles without loading Los (MTem) were adopted in this study. As shown in Figure 1g, MTem eliminated 72.9% of hydrogen peroxide (H_2_O_2_), one of the most common endogenous ROS types, at a concentration of 100 µg mL^−1^ of Tem in 15 min, which was almost close to that of free Tem with 78.9%.

### 2.2 In Vitro ROS‐Scavenging and Anti‐Inflammatory Activities of MTem/Los

Both corneal epithelial cells and macrophages play crucial roles in DED development. Specifically, corneal epithelial cells are core components of the innate immune system and respond directly after exposure to adverse environments. Macrophage is an important phagocytic APC, and the activated ones can secrete proinflammatory cytokines and chemokines as well as further induce a subsequent adaptive response.^[^ ^41^ ^]^ Therefore, both the cells are the target cells in DED management, and regulation of the intracellular DED‐related pathways can alleviate DED‐induced cell death and corneal tissue injury.

**Figure 2:**
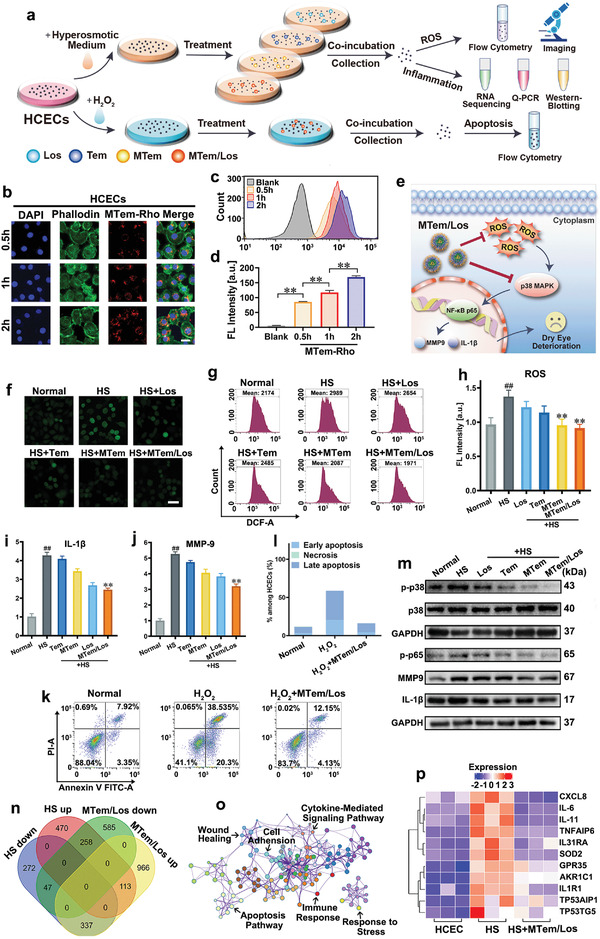
Internalization, anti‐ROS, anti‐inflammatory, and anti‐apoptosis effects and transcriptome analysis in HCECs. a) Scheme of the experimental procedures to evaluate oxidative stress, inflammation, and apoptosis in HCECs. b) Representative fluorescence images of time‐dependent cellular uptake behaviors of MTem‐Rho. Green: Phalloidin; Blue: DAPI; Red: MTem‐Rho signal. The scale bars are 25 µm. c) Flow cytometric curves of internalization of MTem‐Rho (Rho concentration: 10 mm equivalent to those of free Rho) for 0.5, 1, and 2 h into HCECs. d) Quantitative analysis of fluorescent intensity according to the flow cytometry. Data are presented as mean ± SD; n = 3. **p < 0.01. e) Schematic diagram of the proposed mechanism of the inhibition of intracellular signaling pathways and MTem/Los action targets. f) Representative ROS images of HCECs after different treatments using DCFH‐DA staining. The scale bar is 50 µm. g) Flow cytometer diagram of DCF of different treatments. h) Quantitative analysis of the average fluorescent intensity of ROS according to the flow cytometer. RNA levels of i) IL‐1*β* and j) MMP‐9 of HS (500 mOsM L^−1^)‐induced HCECs treated with different groups for 24 h, analyzed by Q‐PCR. Data were presented as mean ± SD; *n*  =  3. ^##^ *p* < 0.01 versus the Normal group and **p < 0.01 versus the HS group. k) Flow cytometry analysis of H_2_O_2_ (400 µm for 2 h)‐induced apoptosis in HCECs after various treatments. l) Proportions of early apoptosis, late apoptosis, and necrosis in HCECs according to flow cytometric results. m) Western Blotting images of p38 MAPK, p‐p38 MAPK, and GAPDH protein expressions (HS [500 mOsM L^−1^] induced for 2 h), NF‐*κ*B p‐p65, MMP‐9, IL‐1*β*, and GAPDH protein expressions (HS [500 mOsM L^−1^] induced for 24 h) of HCECs. n) Venn diagram demonstrating the intersection set of DEAS and differentially expressed genes (DEG). o) Functional analyses from MTem/Los‐associated DEAS events in HCECs, including gene ontology (GO) and KEGG. p) Heat maps of significantly down‐regulated genes in the HCECs after MTem/Los treatment (fold change ≥ 1.5 and *p* < 0.05).

**Figure 3:**
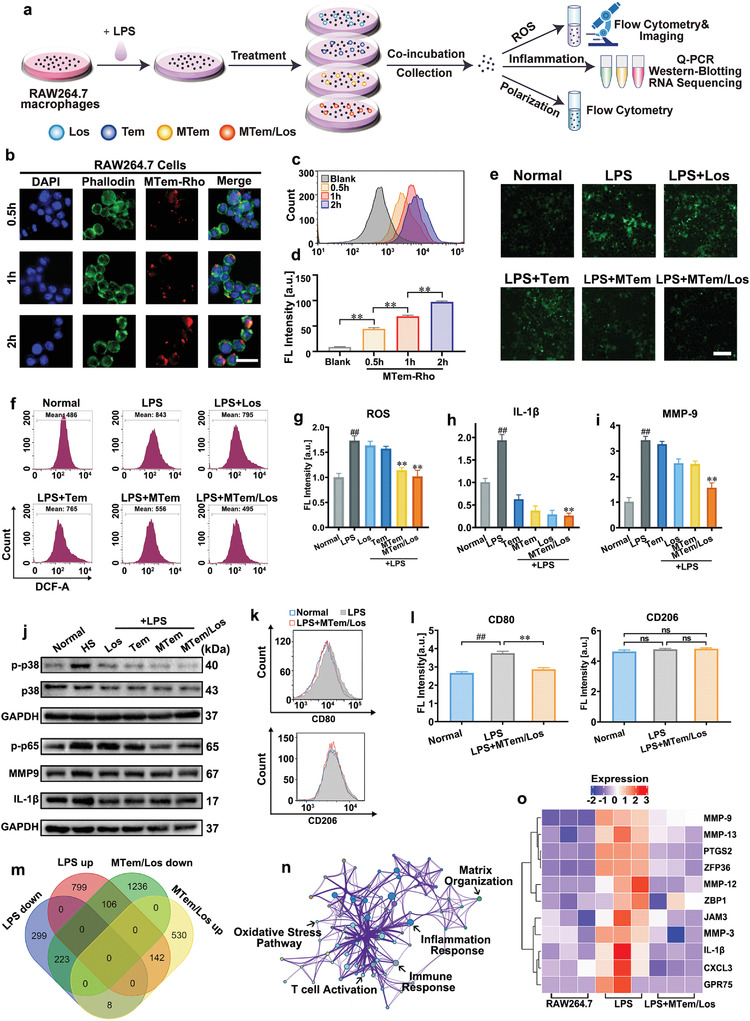
Internalization, anti‐ROS, anti‐inflammation, and anti‐polarization effects and transcriptome analysis in RAW264.7 cells. a) Scheme of the experimental procedures to evaluate oxidative stress, inflammation, and polarization in RAW264.7 cells. b) Representative fluorescence images of time‐dependent cellular uptake behaviors of MTem‐Rho (Rho concentration: 10 mm equivalent to those of free Rho) or 0.5, 1, and 2 h into RAW264.7 cells. Green: Phalloidin; Blue: DAPI; Red: MTem‐Rho signal. The scale bars are 25 µm. c) Flow cytometric curves of internalization of MTem‐Rho. d) Quantitative analysis of fluorescent intensity according to the flow cytometry. Data are presented as mean ± SD; n = 3. **p < 0.01. e) Representative ROS images of RAW264.7 cells by DCFH‐DA staining after different treatments. The scale bar is 50 µm. f) Flow cytometer diagram of DCF of different treatments. g) Quantitative analysis of the average fluorescent intensity according to the flow cytometer. RNA levels of h) IL‐1*β* and i) MMP‐9 in LPS‐induced RAW264.7 cells treated with different treatments after 24 h, analyzed by Q‐PCR. j) Western Blotting images of p38 MAPK, p‐p38 MAPK, GAPDH protein expressions (LPS induced for 1 h), NF‐*κ*B p‐p65, MMP‐9, IL‐1*β*, and GAPDH protein expressions (LPS induced for 24 h) in RAW264.7 cells. k) CD80 and CD206 flow cytometry curves of LPS‐induced RAW264.7 cells. l) Corresponding flow cytometric analysis of CD80 and CD206 in RAW264.7 cells. Data were presented as mean ± SD; n = 3. ns: not significant, p > 0.05, ##p < 0.01 versus the Normal group, and **p < 0.01 versus the LPS group. m) Venn diagram demonstrating the intersection set of DEAS and DEG. n) Functional analyses from MTem/Los‐associated DEAS events in RAW264.7 cells, including GO and KEGG. o) Heat maps of significantly down‐regulated genes in the RAW264.7 cells after MTem/Los treatment (fold change ≥ 1.5 and *p* < 0.05).

The cellular internalization of the micelles in human corneal epithelial cells (HCECs) and RAW264.7 cells was first investigated using rhodamine B (Rho)‐labeled MTem micelles (MTem‐Rho). As shown in **Figures** **2**b and **3**b, time‐dependent fluorescence signals were detected in HCECs and RAW264.7 cells after incubation with MTem‐Rho, and the strong intracellular fluorescence appeared within only half an hour, indicating an exceedingly quick and a large quantity of cellular uptake of the micelles. Moreover, MTem‐Rho transferred and gathered in the cytoplasm of HCECs, where the inhibition of ROS and p38 MAPK took place. The same performance was also found in RAW264.7 cells. Flow cytometry further confirmed the time‐dependent cellular internalization profile of MTem‐Rho in HCECs and RAW264.7 macrophages (Figure 2, 3). These results suggest MTem‐Rho could be internalized rapidly and effectively in vitro, which is beneficial for ocular drug delivery for DED treatment with a relatively short precorneal retention time.

In DED, corneal epithelial cells and the resident macrophages underneath are frequently exposed to ROS attacks and simultaneously play a vital role in inflammatory cytokine production. Accordingly, the effects of ROS elimination and inflammation inhibition of MTem/Los in both cells were investigated. Since hyperosmolarity is one of the core characterizations of DED, 500 mOsm L^−1^ level of HS medium was utilized for inflammation inducement and ROS generation in HCECs (Figure 2a).^[^ ^16^, ^42^ ^]^ Meanwhile, Lipopolysaccharide (LPS) stimulation was adopted in RAW264.7 cells for its comprehensive verifications in oxidative stress stimulation and inflammatory induction (Figure 3a).^[^ ^43^ ^]^ As shown in the proposed mechanism (Figure 2e), MTem/Los possibly suppressed DED deterioration via scavenging of ROS and inhibiting downstream p38 MAPK and NF‐*κ*B p65 activation.

The cellular ROS levels were assessed by 2″,7″‐dichlorofluorescein diacetate (DCFH‐DA) assay through fluorescence microscope observation (Figures 2f and 3e) and flow cytometer analysis (Figures 2g and 3f). As expected, the intracellular ROS levels of HCECs exhibited an apparent increase with a 1.4× elevation of 2″,7″‐dichlorofluorescein (DCF) fluorescein intensity compared to the Normal group upon exposure to HS (Figure 2h). The treatment of Los, Tem, and MTem achieved moderate ROS inhibition effects (11.2%, 16.8%, and 30.2% reductions, respectively), while MTem/Los exhibited the most effective suppression of ROS generation with enormous significance (34.0% reduction). Similarly, RAW264.7 cells exposed to LPS generated 1.7× higher ROS than the Normal group (Figure 3g). Los and Tem slightly reduced ROS levels (decreased by 5.7% and 9.2%, respectively), while the effect was more significant (decreased by 34.0%) in the MTem group. MTem/Los lowered ROS production most efficiently with a 41.2% reduction.

Thereafter, the anti‐inflammatory effects were assessed by Quantitative PCR (Q‐PCR) and Western Blotting. As shown in Figure 2i,j, the RNA levels of IL‐1*β* and MMP‐9 in HCECs were noticeably elevated by factors of 5.2× and 4.3× respectively after HS stimulation. Though the efficacy of Tem was limited, its micelles MTem moderately enhanced the suppression effects on both IL‐1*β* and MMP‐9 RNAs by 15.9% and 16.7%, respectively, compared to the Tem group. Los also showed effective inflammation inhibition (26.9% and 38.4%, respectively, in comparison with the HS group). Notably, the most distinct reduction was from the treatment of MTem/Los, down‐regulating to 39.0% and 43.4% in IL‐1*β* and MMP‐9 expression. Analogously, 1.8‐fold IL‐1*β* and 3.4‐fold MMP‐9 were induced by LPS in RAW264.7 cells (Figure 3h,i). Compared to relatively slight effects in the Los and Tem groups, the MTem group showed greater eliminations in inflammatory cytokines, while the MTem/Los group exhibited the strongest effects with decreases of 70.4% in IL‐1*β* and 54.7% in MMP‐9. Western Blotting was then carried out to further verify the anti‐inflammation effects of MTem/Los at the protein level (Figures 2m and 3j). Investigation of phospho‐p38 (p‐p38) MAPK was first conducted, and the protein expressions of p‐p38 MAPK in both cell lines were most inhibited by MTem/Los while the level of p38 MAPK remained the same level, explained as that Los was a p38 MAPK inhibitor, and the upstream oxidative stress scavenger Tem could enhance the force.^[^ ^44^ ^]^ Next, the downstream NF‐*κ*B phospho‐p65 (p‐p65) protein results demonstrated that all groups alleviated to different degrees after stimulation. Therein, MTem/Los showed the maximal decrement, again verifying the enhanced efficiency by dually scavenging ROS and severing the p38 MAPK pathway signal. Then, we examined the protein levels of IL‐1*β* and MMP‐9 in HCECs and RAW264.7 cells. Slightly different results from the above‐proved RNA level were observed in protein expression as there were nearly no obvious variations in the Los nor the Tem groups in HCECs. In contrast, reductions of IL‐1*β* and MMP‐9 were found in the MTem/Los group, which substantiated the effective therapeutic efficacy of collaborative therapy at the level of translation. In RAW264.7 cells, the decreased tendencies of protein expressions of IL‐1*β* and MMP‐9 were roughly consistent with the previous correspondent RNA results. Image analysis results of protein levels are shown in Figure S9 and S10, Supporting Information. The results demonstrated that our fabricated MTem/Los could both reduce ROS production and hinder the activation of p38 MAPK, thus downregulating relative signal paths and downstream genes in a more effective way.

Since the apoptosis of corneal epithelial cells is commonly believed to have a critical role in the vicious cycle and a typical adverse outcome in DED progression, the modulation of apoptosis in HCECs by MTem/Los was further studied. Two hour of incubation with 400 µm H_2_O_2_ severely elicited around 20.3% of early apoptosis and 38.5% of late apoptosis in HCECs, whereas the rates in the Normal group were only 3.35% and 7.92%, respectively (Figure 2k,l). Surprisingly, MTem/Los impeded apoptosis to an extreme degree, with an early apoptosis rate of 4.13% and late apoptosis rate of 12.1%, which was close to the normal state, showing the robust protective effect towards apoptosis in epithelial cells and indicating potential in vivo therapeutic efficacy when exposed to excessive environmental adverse stresses.

Apart from the inflammation of epithelial cells, the stimulation of macrophages also initiates the immunoinflammatory pathway in DED vicious cycle. Activated macrophages manifest a heterogeneous cell population (M1, M2, and others), and the polarization towards M1 has a more pro‐inflammatory tendency, while those of M2 acquire the quality of inflammation resolution. Previous studies have reported that there would be a polarization towards the inflammatory phenotype in corneal and conjunctival resident macrophages under desiccating stress conditions.^[^ ^45^ ^]^ To confirm the impact of MTem/Los on macrophage polarization, we utilized LPS to stimulate inflammation with CD80 antibodies labeled as M1 macrophages and CD206 antibodies labeled as M2 macrophages, following measuring the fluorescent intensity of both macrophages through flow cytometry. As Figure 3k,l show, LPS stimulation significantly increased M1 macrophages while maintaining M2 ones, indicating a deteriorative inflammatory response. However, the subsequent addition of MTem/Los effectively decreased M1 macrophages so far as to nearly recover to the normal state. Less inflammation tendency on macrophages could then alleviate DED due to the decreased level of inflammatory cytokines and antigen presentation ability.

In order to comprehensively explore the intracellular mechanism after MTem/Los treatment, whole transcriptome RNA sequencing in both cell lines was performed. The transcription of 16 886 genes, in total, was examined. Thereinto, 258 genes were elevated with HS pretreatment and subsequently decreased by MTem/Los treatment (Figure 2n), while 106 genes in the LPS group firstly increased and then went down in the MTem/Los group (Figure 3m). Those corresponding genes were further assessed with the Kyoto Encyclopedia of Genes and Genomes (KEGG) pathway enrichment analysis of differentially expressed alternative splicing (DEAS) with the most significantly enriched pathways shown in Figures S11 and S12, Supporting Information. In addition, functional and pathway enrichment analysis was conducted to further analyze and visually display the interactions among signal pathways (Figures 2o and 3n). The remarkably enriched terms of the two cell lines contained “inflammatory response”, “cytokine signaling in immune system”, “apoptosis signaling pathway”, and “oxidative stress and redox pathway”, which are related to inflammation, immune response, apoptosis, and oxidative stress, respectively. More specifically, inflammatory‐related (e.g., IL‐1*β*, IL‐6, TNFAIP6), chemotaxis‐related (e.g., MMP‐3, MMP‐9, CXCL8), oxidative stress‐related (e.g., SOD2), and apoptosis‐related genes (e.g., TP53TG5, TP53AIP1) were down‐regulated by MTem/Los treatment (Figures 2p and 3o). These results demonstrate that MTem/Los manifest its excellent effects via widespread influences under DED threats in vitro.

Altogether, MTem/Los effectively inhibited ROS generation and the p38 MAPK site, decreased the downstream inflammatory‐related and chemotaxis‐related expressions and cell apoptosis, and reverted the proinflammatory phenotypic switching of macrophages, all of which are involved in the pathogenesis of DED. This encouraged us to further proceed with its therapeutic outcome in vivo.

### 2.3 In Vivo Therapeutic Effects on DED

**Figure 4:**
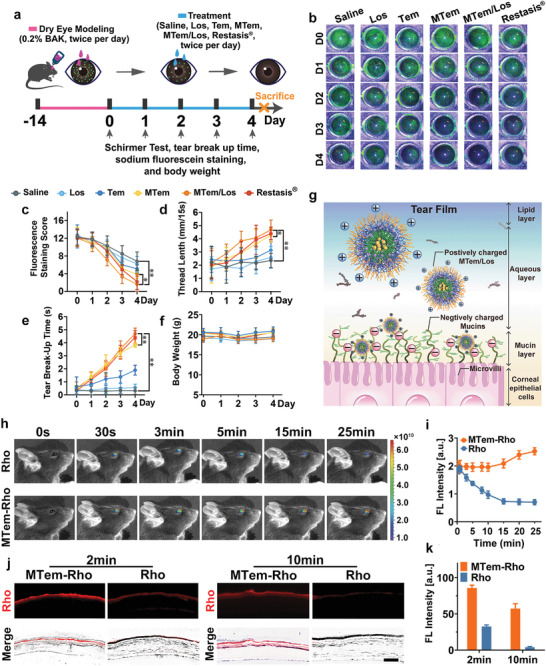
The therapeutic benefits of various therapies on BAK‐induced DED and the preocular retention assessment of MTem on dry eye models. a) Scheme of in vivo DED modeling and treatment. The dry eye models were established by instillation of 5 µL of 0.2% w/v BAK eye drops twice per day for 2 weeks. Then the mice received topical instillation of 10 µL of 0.9% w/v saline, Los (15.6 µg kg^−1^), Tem (2.5 mg kg^−1^), MTem (Tem: 2.5 mg kg^−1^), MTem/Los (Tem: 2.5 mg kg^−1^, Los: 15.6 µg kg^−1^), and Restasis twice per day, respectively. The therapeutic effects were evaluated and recorded every day during the therapy process. b) Representative fluorescein sodium staining images, c) corresponding staining scores, d) wetted phenol thread lengths by the Schirmer test, e) TBUT, and f) daily body weight records of the DED mice treated with different therapies throughout the 4‐day treatment process. Data are presented as mean ± SD; *n*  =  10. *p < 0.05, **p < 0.01. g) Schematic diagram of the electrostatic interaction between positively charged MTem/Los and negatively charged mucins in tear film on the ocular surface. h) Real‐time in vivo fluorescence imaging of free Rho (10 mm) and MTem‐Rho (Rho concentration: 10 mm equivalent to those of free Rho) on eyes of anesthetic mice at different time points (0, 0.5, 3, 5, 15, and 25 min). i) Quantitative analysis of fluorescein intensity changes on eyes of anesthetized mice after administration of Rho or MTem‐Rho at different time points. j) Fluorescent images of ocular surface frozen sections after administration with Rho or MTem‐Rho on mice under conscious states for 2 or 10 min. The scale bar is 50 µm. k) Quantitative analysis of average fluorescein intensity of ocular surface frozen sections after administration of Rho or MTem‐Rho for 2‐ or 10‐min. Data are presented as mean ± SD; *n*  =  3.

To evaluate the therapeutic outcome of MTem/Los on DED, an experimental mouse model of dry eye, induced by topical administration of high‐dose benzalkonium chloride (BAK), was first constructed. BAK is a widely‐used preservative added to most ocular formulations and can induce dry eye symptoms mainly through causing damage to corneal epithelial cells and accelerating tear film breakdown and evaporation, all of which are accompanied by excessive ROS generation. Accordingly, the mice were administered with 14‐day‐lasting 0.2% BAK eye drops twice per day and subsequently received different treatments, including Saline, Los, Tem, MTem, MTem/Los, or Restasis twice a day for 4 days (**Figure** **4**a). These experimental mice had typical clinical symptoms such as defects on the corneal surface and tear secretion deficiency, indicating the successful establishment of dry eye models.^[^ ^46^ ^]^ Since the corneal epithelium of normal eyes barely has any defects with negligible fluorescein spots but that of dry eyes results in defects that exhibit positive fluorescein staining spots, the fluorescein staining can be used for the evaluation of the therapeutic effects on DED. After various topical ocular treatments, the fluorescein staining images of the cornea were monitored photographically by slit‐lamp microscopy, and relevant fluorescent vital staining scores by a 16‐point scale were then recorded. As shown in Figure 4b,c, the Saline group held strong fluorescence with a staining score of >6.0 continuously for 4 days, implying severe dry eye symptoms. Monotherapies, including Los, Tem, and MTem therapies, could alleviate the dry eye symptom as characterized by reduced fluorescence. Nevertheless, the residual fluorescein staining on the corneal, with a staining score of more than 5.0, implied that the dry eye‐induced corneal epithelial defects were not fully cured on day 4 after monotherapy treatment. Excitingly, after 4‐day treatment of MTem/Los, the fluorescence on the cornea of mice decreased gradually with a final average staining score of 1.6, substantiating a superior therapeutic outcome. It was worth noting that MTem/Los possessed better therapeutic benefits than commercial Restasis. This is likely because the therapeutic target of Restasis is the adaptive immune response, more specifically T cell, and Restasis generally takes a longer period, from weeks to months, to take effect.^[^ ^47^ ^]^ Apart from fluorescein staining, diagnosis of DED symptoms also included tear‐related assessment such as a Schirmer test, a classic measurement of tear secretion amount, and tear break‐up time (TBUT), a common clinical index for tear stability evaluation.^[^ ^48^ ^]^ The Schirmer test (Figure 4d) results showed the average tear volume of normal mice was 4.81 ± 0.72 mm, while that of mice with DED dramatically reduced to 2.36 ± 0.52 mm, indicating a marked reduction in tear secretion and successful initiation of dryness in the dry eye mouse model. Los had little effect (2.59 ± 0.77 mm) on dry eye therapy as a result of its insolubility and short retention time in tears, leading to extremely low bioavailability and treatment outcome. Tem, and its cationic micelle formation, MTem, had higher Schirmer test results of 3.24 ± 0.65 and 4.27 ± 0.50 mm, respectively. MTem/Los, as expected, exerted an optimal treatment effect with a tear volume of 4.62 ± 0.69 mm, which was comparable to commercial Restasis (4.35 ± 0.51 mm) and even almost the closest to normal tear volume. The TBUT test (Figure 4e) was further performed to study the tear film stability largely dependent on the mucous secretions.^[^ ^49^ ^]^ Similarly, a dramatic reduction in TBUT (0.3 ± 0.26 s) was observed in the Saline group. Los had few effects on dry eye therapy with TBUT, measuring only 0.6 ± 0.25 s. The Tem (1.9 ± 0.36 s), MTem (3.8 ± 0.17 s), Restasis (4.4 ± 0.48 s), and MTem/Los (4.7 ± 0.40 s) groups exhibited better tear film stability and tear production with a statistically significant difference with saline, demonstrating the maximum therapeutic benefit of MTem/Los.

### 2.4 In Vivo Underlying Mechanisms

The inspiring clinical therapeutic efficacy of MTem/Los encouraged us to explore its precorneal retention ability. We adopted the real‐time in vivo fluorescence imaging technique to detect and track the distribution and retention of MTem‐Rho on the eyes of dry eye model mice.^[^ ^50^, ^51^ ^]^ As shown in Figure 4h, the MTem‐Rho group had almost the same fluorescence intensity as the Rho group after administration for the first 30 s. After that, the fluorescence of the Rho group constantly diminished, while that of the MTem‐Rho group remained steadily elevated. Concretely, the fluorescence intensity of the MTem‐Rho group was ≈2.8‐ and 3.6‐fold that of the Rho group at 15 and 25 min after instilling, respectively (Figure 4i). The intriguing result of gradual increased MTem‐Rho fluorescence intensity on the cornea is assumed to be the rapid evaporation of tears in vivo imaging system machine (37 °C), together with hardly any newly secreted tears formed under anesthesia, leading to the concentrated fluorescent dye.^[^ ^52^ ^]^ For the sake of eliminating this distraction, mice without receiving anesthesia treatment were adopted, and their corneal tissues were harvested to study the precorneal retention of MTem consciously (Figure 4j). It suggested that the fluorescence of free Rho on the corneal was weak, while that of MTem‐Rho was 2.6× stronger after 2 min post‐administration. After 10 min of administering the eye drops, the red fluorescence of free Rho almost vanished, but surprisingly, the strong red fluorescence of MTem‐Rho was still on the corneal surface. Through image analysis, we found that the resident average fluorescent intensity of 10 min was up to 70.0% of that of 2 min (Figure 4k). This extraordinary improvement in precorneal retention time can be ascribed to the strong electrostatic interaction between cationic MTem and negatively charged mucin distributed on the cornea as illustrated in Figure 4g. This peculiar property could enhance the retention time of MTem on the ocular surface and guarantee its prolonged and intimate contact with corneal cells, making MTem an ideal nanocarrier for ocular surface diseases like DED.

**Figure 5:**
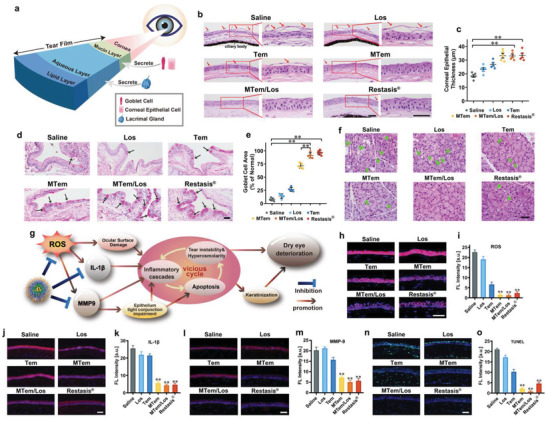
Representative cell morphology and structure of the cornea, amplifying corneal epithelium, lacrimal gland, and conjunctival epithelium after 14 days of various treatments in DED modeling. a) Illustration of three‐layer tear construction and their corresponding main supplying organs. b) Representative H&E staining images of the cornea and corresponding amplifying corneal epithelium, d) representative conjunctival PAS staining images, and f) lacrimal gland H&E staining images. Red arrow: desquamated corneal epithelium. Green triangle: lymphocytes infiltration and structural disorder in lacrimal gland tissue. Black arrow: goblet cells. The scale bar is 50 µm. c) Quantitative analysis of the corneal epithelial thickness. e) Quantitative analysis of goblet cell area percentage compared with Normal group in the conjunctiva. Data were presented as mean ± SD; *n* = 5. g) Schematic diagram of the mechanism of DED deterioration defense conducted by MTem/Los. h) Evaluations of oxidative stress (ROS), inflammatory factors (j) IL‐1*β* and l) MMP‐9), and n) apoptosis (TUNEL) by immunostaining on the corneal epithelial cells in the mice eyes after 14 days of diverse therapies for DED. The scale bars are 50 µm. Quantitative analysis of total fluorescence Intensity of i) ROS‐, k) IL‐1*β*‐, m) MMP‐9‐, and o) Tunel‐positive areas. Data were presented as mean ± SD; *n* = 3. **p < 0.01 versus the Saline group.

We then conducted histomorphology assessments to investigate the structural and morphological changes in ophthalmic organs after various treatments. The tear film has a three‐layered structure, namely mucus, aqueous, and lipid layer (from inside to outside), secreted by multiple ocular tissues and cells as illustrated in **Figure** **5**a. Corresponding damages to these tissues and cells would lead to accelerated tear film breakdown and evaporation, resulting in an imbalance in tear film homeostasis.

The overall corneal structure and cell morphology were first evaluated by Hematoxylin&Eosin (H&E) staining. The normal cornealcells were densely and neatly arranged without inflammatory cells infiltration (Figure S13, Supporting Information). However, the corneal superficial epithelial layers turned disrupted and desquamated, approximately half thinning after DED modeling as shown in Figure 5b. The number of epithelial cells decreased, and the morphology turned significantly irregular. Moreover, its corneal stromal layer became thicker and looser, filled with excessive vacuoles and infiltrated inflammatory cells. These changes in corneal cell morphology and structure were in line with the reported dry eye syndrome.^[^ ^53^ ^]^ After treatment of MTem, MTem/Los, and Restasis, the corneal cell morphology and structure recovered to a large extent, while those of Los and Tem groups had limited improvement. The corneal epithelial thickness results of the Normal (31.6 ± 2.06 µm) (Figure S13, Supporting Information), Saline (18.2 ± 2.14 µm), Los (23.2 ± 2.64 µm), Tem (26.8 ± 2.48 µm), MTem (33.2 ± 3.43 µm), MTem/Los (33.0 ± 2.61 µm), and Restasis (33.4 ± 3.26 µm) groups (Figure 5c) also confirmed the excellent therapeutic outcomes of MTem/Los.

The mucus layer, the innermost of the three tear layers, is mainly produced by goblet cells. Goblet cells are specialized cells that secrete mucins to lubricate the ocular surface, and its loss is a key feature in mice with DED.^[^ ^54^ ^]^ As shown in Figure S14, Supporting Information, the periodic acid‐Schiff (PAS)‐positive goblet cells are abundantly presented in the conjunctival fornix of the normal cornea. However, after DED modeling, the goblet cells in the Saline group appeared deflated (Figure 5d), and the average area of stained cells in the conjunctiva significantly reduced to 7.78% compared with the Normal group (Figure 5e). Moreover, these goblet cells exhibited a small and shriveled morphology, displaying little or no clustering, and were sparsely distributed along the conjunctival epithelium, leading to mucin deficiency and tear film instability. Nevertheless, the area of goblet cells in the MTem/Los group had been restored markedly to 92.3% compared to the Normal group, which was more effective than other treatment groups, with a statistically significant difference (*p* < 0.01). This is possibly due to the effective inhibition of cytokine productions from innate immune cells that could trigger the apoptosis of goblet cells. Another noteworthy observation was that the morphology and area of goblet cells in the commercial Restasis group recovered to the nearly normal state (97.7%). This result is consistent with previous clinical data,^[^ ^55^ ^]^ showing that topical Restasis might treat DED through increasing goblet cell density by suppressing goblet cell loss‐related immune cytokines produced by T cells.

The aqueous layer, as the most abundant component of the tear film, is primarily secreted by the lacrimal gland. The lacrimal gland mainly consists of acinar cells (about 80%) and infiltrating immune cells (such as lymphocytes). The acini in the healthy gland exhibited uniformity in size, neatness in arrangement, and normality in morphology. Specifically, the cellular nuclei were equally shaped and located at the base of the cell surrounded by homogeneous cytoplasm (Figure S15, Supporting Information). After the dry eye modeling, the lacrimal gland structure turned chaotic. Numerous acini sized unevenly, and its arrangement became disorderly, which displayed a concrete manifestation of atrophy and fusion, accompanied by intracellular vacuoles and infiltrated lymphocytes externally (Figure 5f). Surprisingly, treatment of MTem/Los and MTem dramatically saved the structural disorder and recovered very closely to the healthy condition with the neat arrangement, while Los or Tem treatment could not. More notably, morphologies in MTem/Los and MTem groups were observed to be better formed than that attained in the commercial Restasis group, which retained the vacuoles and gland fusion problem.

BAK, as a strong preservative, can easily attack lipid constructions of the lacrimal surface, accompanied by an increase in large amounts of excess ROS. However, as we previously proved, MTem/Los exhibited robust ROS elimination capability, which could potentially protect lipid layers through effective ROS clearance.^[^ ^56^ ^]^

Given the three‐pronged protection of tear film, MTem/Los had been demonstrated to effectively promote tear stabilization and increase the liquid products in the dry eye mouse model, which corresponds with the previously proven Schirmer test and TBUT results. All these results confirm that MTem/Los can critically restore the corneal cell morphology and structure as well as recover the tear secretion function of dry eye models.

To further investigate the underlying mechanisms behind the recovery of corneal structure and cell morphology of MTem/Los, the anti‐oxidative and anti‐inflammatory levels as well as anti‐apoptosis and anti‐keratinization effects in the corneal tissue after different treatments were further assessed by immunohistochemistry, including ROS, IL‐1*β*, MMP‐9, TUNEL, Ki67, and K10 assays.

In DED, the elevation of ROS and proinflammatory cytokines such as IL‐1*β* and MMP‐9 can bring out damage to the cornea, especially to the epithelial layer.^[^ ^57^ ^]^ After DED modeling, indeed, a sharp increase in ROS intensity and elevated expression of IL‐1*β* and MMP‐9 were observed on the corneal surface and in the corneal epithelia, respectively. After 4‐day consecutive treatments, the Los and Tem groups remained high in ROS (Figure 5h), IL‐1*β* (Figure 5j), and MMP‐9 (Figure 5l) expression, but the MTem, MTem/Los, and Restasis groups exhibited effective inhibition, especially MTem/Los, achieving the most effective suppressions (89.7% reduction of ROS, 80.8% reduction of IL‐1*β*, and 71.8% reduction of MMP‐9, respectively) according to the total fluorescence intensity quantitative analyses (Figure 5i,k,m). Notably, the residual ROS detected on the corneal surface of MTem, and MTem/Los group were less than that of Restasis due to the additionally designed characteristic of ROS scavenging compared with Restasis's humble anti‐inflammatory property. In the IL‐1*β* assessment, a similar result was found that the IL‐1*β* suppression ability of MTem/Los was superior to that of the Restasis group. On the one hand, it verified the powerful effect on DED treatment by inhibiting inflammation. On the other hand, the coincident reduced tendency validated our putative conjecture that the decline of inflammation could be strengthened by ROS reduction. Unignorably, those positively charged carriers which enhanced the adhesion to the cornea and thus prolong the retention time on the ocular surface, consequently enhancing ROS elimination, contributed greatly to the improving efficacy compared with commercial eye‐drops.

DED can generate a large number of ROS via various pathogenesis.^[^ ^58^ ^]^ Therefore, the efficient elimination of excess ROS is alternated as a powerful approach in DED management. This explains the exceptional performance of MTem and MTem/Los in tear, goblet cell, and lacrimal gland as mentioned above. As a typical and significant proinflammatory cytokine, IL‐1*β* activates innate response, amplifies inflammatory cascades, and stimulates APCs that can activate a further adaptive immune response, all of which result in deterioration of the DED process. MMP‐9 can result in corneal barrier dysfunction and surface irregularity, leading to cell apoptosis. It can also initiate inflammatory cascades by cleaving pro‐cytokines, stimulating other extracellular proteins (receptors, growth factors, and adhesion molecules),^[^ ^59^ ^]^ and APCs like macrophages. The elevation of MMP‐9 is vitally and closely related to dry eye syndrome, and its detection has been utilized as a novel means in the diagnosis of DED.^[^ ^59^ ^]^ The powerful MMP‐9 elimination in MTem/Los group explains why the minimum damage occurred in corneal epithelial cells (Figure 5b) with the fewest green spots observed under the slit lamp (Figure 4b).

Specifically, the proliferation and apoptosis levels in the cornea after different therapies were determined by Ki67 and TUNEL assay, respectively. Consistent with previous studies,^[^ ^20^, ^60^ ^]^ numerous TUNEL‐positive cells(Figure 5n) and few Ki67‐positive cells (Figure S16, Supporting Information) were detected, indicating high apoptosis, low proliferation, and cell injury in BAK‐induced DED models. Los and Tem therapy had fewer effects with high cell apoptosis levels in comparison to the Saline group. However, MTem, MTem/Los, and Restasis groups exerted efficient inhibition (89.7%, 96.1%, and 78.1%, respectively) of apoptosis by fluorescence intensity analysis (Figure 5o). Particularly, the MTem/Los group had the most Ki67‐positive cells detected than other groups, reflecting its superior cell repairability, which was consistent with the therapeutic benefits demonstrated above.

Keratinization, also known as squamous metaplasia, is one of the most common ocular surface pathological changes that can progress to corneal opacification and vision loss in dry eye syndrome,^[^ ^61^ ^]^ which can be evaluated by K10 assay. As shown in Figure S17, Supporting Information, the Saline and Los, as well as Tem groups, exhibited strong red fluorescence with high K10 expression, revealing apparent squamous metaplasia. Yet the MTem, MTem/Los, and Restasis groups showed significant protective effects against BAK‐induced squamous metaplasia. Thereinto, the MTem/Los group had the least K10 expression, indicating the strongest inhibition effect on squamous metaplasia, which might be originated from alleviatied inflammation as squamous metaplasia is an end‐stage manifestation of ocular inflammation.^[^ ^62^ ^]^ All the immunohistochemistry results above suggest that our strategy of combined oxidative stress and inflammation suppression may be more efficient compared to the commercial monotherapy immunosuppressant when dealing with DED. The mechanism of DED deterioration prevention conducted by MTem/Los is schematically summarized in Figure 5g.

Altogether, MTem/Los heavily restored the ocular surface structures morphologically through the pathway of inhibiting ROS overexpression, inflammation response, cell apoptosis, and squamous metaplasia, thereby realizing an effective strategy for DED therapy.

### 2.5 Safety Evaluation

Given the prominent therapeutic results, the current anti‐oxidative micelles loaded with anti‐inflammatory drugs in DED therapy hold a bright future for clinical translation. On this account, it is necessary to evaluate the biosafety of MTem/Los. Specifically, in vitro and in vivo toxicity experiments, including cytotoxicity, body weight, ocular irritation, and histological assessments of mouse cornea and major organs, were carried out in this study. Cell counting kit‐8 (CCK‐8) assay and Live/Dead cell staining were utilized for evaluating the cytotoxicity. The cytotoxicity of MTem/Los was negligible regardless of various doses in HCECs or RAW264.7 cells for 24 h. As is shown in Figures S18 and S19, Supporting Information, the cell viability of MTem/Los was up to 86.1% and 93.9% in HCECs and RAW264.7 macrophages respectively, putting the dosage as high as 1200 µg mL^−1^/100 µg mL^−1^ (Los/Tem). Also, the Live/Dead cell staining results again support that MTem/Los exhibits excellent cytocompatibility with almost no cytotoxicity (Figures S20 and S21, Supporting Information).

As for in vivo safe evaluation, mice in all groups, including Saline, Los, Tem, MTem, and MTem/Los, maintained stabilized body weights and did not manifest observable behavioral abnormalities, irrespective of normal mice or dry eye ones (Figure 4f and Figure S22, Supporting Information). The toxicity of MTem/Los to the cornea was then assessed since it was applied directly on the ocular surface. No abnormal clinical signs, including corneal defects, opacification, tear turbidity, corneal neovascularization, conjunctival hyperemia, or inflammation, were observed in mice eyes with different treatments after administration twice a day for 2 continuous weeks (Figure S23a,b, Supporting Information). Additionally, fluorescein staining showed no distinct corneal epithelial defects in each group (Figure S23c, Supporting Information). Moreover, corneal morphology, integrity, and thickness of the epithelium in each group remained normal after topical instillation, as uncovered by H&E staining (Figure S23d, Supporting Information). Then, gross necropsies and histological analysis on major organs were implemented to evaluate the long‐term toxicity of MTem/Los on the 30th day after treatment. As displayed in Figure S24, Supporting Information, no gross or histopathological abnormalities or lesions in the heart, liver, kidney, spleen, or lung were observed. These data, taken together, reveal that MTem/Los has high biocompatibility and excellent ocular tolerance without obvious in vitro cytotoxicity, in vivo systemic toxicity, or corneal toxicity. It suggests that MTem/Los has acquired the capacity to act as a safe nano‐therapy approach for future clinical application.

## 3 Discussion and Conclusion

Dry eye disease possesses increasing morbidity worldwide and can cause serve ocular damage. However, current therapeutic strategies reveal disadvantages such as short ocular surface retention time, slow action, and ophthalmic complications after long‐term application. These gaps manifest tremendous unmet needs. Therefore, we designed and prepared p38 MAPK inhibitor Los loaded, antioxidant Tem conjugated cationic polypeptide micelle‐based eye drops, MTem/Los, with improved ocular retention time, for highly effective treatment of DED through breaking DED vicious cycle.

Rapid internalization in corneal epithelial cells and macrophages and prolonged ocular surface retention time of MTem/Los are very favorable for DED therapy. ROS plays extensive damage to the ocular surface and diverse points in the cycle while accelerating inflammation progression. At the same time, injury of corneal epithelial cells and stimulation of macrophages are key progressions in the inflammatory pathway. Through dual‐functional execution, MTem/Los strongly suppressed the DED vicious cycle from the initial step via inhibiting overproduced oxidative stress by Tem as well as an intracellular signal pathway at p38 MAPK by Los in epithelial cells and macrophages, which are both core cells of the DED vicious cycle. Notably, in mice with DED, MTem/Los was superior to commercial Restasis efficacy in some aspects, concluding that the strong inhibition of inflammation in early stages is more effective when alleviating DED compared with the immunotherapy. Further applications of these efficient anti‐inflammation micelles could be expanded for other diseases by changing the drug used, and/or drug dosage, and so on.

All in all, we demonstrated that MTem/Los could efficiently rescue DED deterioration through breaking its vicious circle comprising suppressions of inflammation, ROS generation, and apoptosis, with excellent biosafety and ocular tolerance for its future application in the clinic, not merely ocular surface pathologies, but other inflammation‐based diseases.

## 4 Experimental Section

### Preparation of Los‐Loaded Anti‐Oxidative Micelles (MTem/Los)

Los‐loaded anti‐oxidative micelles (MTem/Los) were prepared by the dialysis method. Twenty mg of polypeptide PEG‐*b*‐(Lys‐*graft*‐Tem‐*co*‐Phe) and 1 mg of Los were dissolved in 2 mL DMSO and stirred for 4 h. Two mL of water was then added dropwise and kept being stirred for 4 h. The solution was transferred into a dialysis tube (MWCO 3500), followed by dialyzing against water for 48 h to remove DMSO. Anti‐oxidative micelles without being loaded with Los (MTem) were also prepared using the same procedure.

### In Vitro Los Release Behavior of MTem/Los Micelles

The release rate of Los from MTem/Los micelle was evaluated using the dialysis method. In detail, 2 mL of MTem/Los micelle was added into a dialysis bag (MWCO 3500), then immersed into 8 mL of PBS containing 1% (w/v) Tween 80 with/without 5 µg mL^−1^ lysozyme at 37 °C in a thermostated incubator with constant shaking (200 rpm). At predetermined time intervals, 2 mL of the release medium was drawn and replenished with the corresponding volume of fresh release medium. The Los in the release medium was measured by HPLC.

### Anti‐Oxidative Effects of MTem/Los

The ROS scavenging capability of MTem/Los was assessed by a hydrogen peroxide (H_2_O_2_) assay kit according to previously reported literature with slight modifications.^[^ ^63^ ^]^ Briefly, different concentrations of free Tem (0, 0.01, 0.1, 1, 10, 50, 100, 500 µg mL^−1^) and MTem (concentrations equivalent to those of free Tem) were incubated in 1 mL of 0.01 m phosphate buffer saline (PBS, pH 7.4) containing 400 µm H_2_O_2_ for 30 min. Then residual H_2_O_2_ was determined by a hydrogen peroxide assay kit (S0038, Beyotime), and eliminated H_2_O_2_ was calculated.

### In Vitro Cytotoxicity Assays

Cell counting kit‐8 (CCK‐8) and Live/Dead assays were utilized for evaluating the cytotoxicity as follows:^[^ ^36^ ^]^ For CCK‐8 assay, both HCECs and RAW264.7 cells were planted first in 96‐well plates overnight at a density of 2 × 10^4^ and 4 × 10^4^ cells per well, respectively. Different samples, including Los, Tem, MTem, and MTem/Los (with a final concentration of Los/Tem: 0 ng mL^−1^/0 µg mL^−1^, 0.6 ng mL^−1^/0.05 µg mL^−1^, 6 ng mL^−1^/0.5 µg mL^−1^, 60 ng mL^−1^/5 µg mL^−1^, 600 ng mL^−1^/50 µg mL^−1^, and 1200 ng mL^−1^/100 µg mL^−1^), were then added. After incubation for 24 h, 100 µL of CCK‐8 solution (Dojindo) was added to each well according to the manufacturer's protocol. The absorbances were then measured by a microplate reader (iMark Microplate Absorbance Reader, Bio‐Rad) at 450 nm. Results were expressed as a percentage of the control. For Live/Dead assay, HCECs and RAW264.7 cells were cultured in six‐well plates overnight at a density of 4 × 10^5^ and 1 × 10^6^ cells per well, respectively. MTem/Los (with a final concentration of Los/Tem: 0 ng mL^−1^/0 µg mL^−1^, 0.6 ng mL^−1^/0.05 µg mL^−1^, 6 ng mL^−1^/0.5 µg mL^−1^, 60 ng mL^−1^/5 µg mL^−1^, 600 ng mL^−1^/50 µg mL^−1^, and 1200 ng mL^−1^/100 µg mL^−1^) was added to the complete medium and subsequently incubated for 24 h. Live/Dead Viability/Cytotoxicity Kit (Thermo Fisher) was then added and incubated for 30 min at 37 °C, followed by being rinsed three times with PBS and photographed under a fluorescence microscope (DMi8, LEICA).

### Evaluation of Cellular Internalization Profiles of MTem/Los

Fluorescence imaging and flow cytometric analysis were conducted to investigate the cellular uptake of the micelles as follows; HCECs and RAW264.7 cells were cultured upon microscope‐cover‐glasses in 24‐well plates overnight at a density of 1 × 10^5^ and 3 × 10^5^ cells per well, followed by incubation of rhodamine B‐labeled MTem micelles (MTem‐Rho) (rhodamine B concentration: 10 mm equivalent to that of free rhodamine B) for 0.5, 1, and 2 h, respectively. Afterward, cells were rinsed and added with Phalloidin (C1033, Beyotime) for 90 min at room temperature followed by DAPI staining (Sigma). After 3 times of wash, cells on glasses were covered with antifade mounting medium (H‐1000 VECTASHIELD, Vector) and observed under a fluorescence microscope. For flow cytometric analysis, cells were directly seeded in the blank wells without cover glasses. After incubation with MTem‐Rho for 0.5, 1, and 2 h, cells were removed and collected by cell scrapers. Samples were further measured quantitatively as the average rhodamine B fluorescence intensity via a flow cytometer (FACSCanto II, BD Biosciences). Data were exported and analyzed by FlowJo software.

### In Vitro Anti‐Oxidative Stress of MTem/Los in Cells

In HCECs, hyperosmotic stress (HS) medium (500 mOsm L^−1^) was applied to generate excess ROS. In RAW264.7 macrophages, Lipopolysaccharide (LPS) (1 µg mL^−1^) was added for ROS stimulation. HCECs and RAW264.7 cells were cultured overnight in 12‐well plates at a density of 1.5 × 10^5^ and 4 × 10^5^ cells per well. The HCECs were pretreated with Los, Tem, MTem, and MTem/Los (Tem: 600 ng mL^−1^, Los: 50 µg mL^−1^ at a final concentration) for 1 h and then switched to HS medium for 24 h. Concerning RAW264.7 cells, similarly, the macrophages were pretreated with Los, Tem, MTem, and MTem/Los (Tem: 600 ng mL^−1^, Los: 50 µg mL^−1^ at a final concentration) for 1 h and then exposed to LPS for 24 h as well. After incubation, cells were rinsed and treated with 2,7‐dichlorofluorescein diacetate (DCFH‐DA) (10 µm) of ROS Assay Kit (S0033S, Beyotime) in serum‐free DMEM for 30 min at 37 °C in the dark. After being washed with PBS for 3 times, fluorescence images were captured under a fluorescence microscope. Through similar procedures, the intracellular ROS generation of collected cells was further measured quantitatively as the average DCF fluorescence intensity via flow cytometer. Data were exported and analyzed by FlowJo software.

### In Vitro Anti‐Inflammatory Effect of MTem/Los in Cells

HS (500 mOsm L^−1^) and LPS (1 µg mL^−1^) were chosen to stimulate the inflammation in HCECs and RAW264.7 cells, respectively. HCECs and RAW264.7 cells were seeded onto six‐well plates at a density of 4 × 10^5^ and 1 × 10^6^ cells per well overnight to allow attachment. The HCECs were pretreated with Los, Tem, MTem, and MTem/Los (Tem: 600 ng mL^−1^, Los: 50 µg mL^−1^ at a final concentration) for 1 h and then exposed to HS for 2 or 24 h, while RAW264.7 macrophages were pretreated with Los, Tem, MTem and MTem/Los (Tem: 600 ng mL^−1^, Los: 50 µg mL^−1^ at a final concentration) for 1 h and then exposed to LPS for 1 h or 24 h as well. Q‐PCR analysis and Western Blotting analysis were then used to investigate the anti‐inflammatory effects of MTem/Los.

### In Vitro Anti‐Apoptosis Activity of MTem/Los in HCECs

In HCECs, H_2_O_2_ (400 µm) was applied to induce apoptosis. HCECs were seeded in a six‐well plate at a density of 4 × 10^5^ cells per well overnight. Cells were preincubated with MTem/Los (Tem: 600 ng mL^−1^, Los: 50 µg mL^−1^ at a final concentration) for 1 h, and then replaced with a medium containing freshly diluted H_2_O_2_ (400 µm) for apoptosis inducement. Both adherent and supernatant cells were harvested with trypsin solution without EDTA (C0205, Beyotime) and washed with PBS once. Apoptotic cells were then identified and quantified by Apoptosis Detection Kit (C1062M, Beyotime) according to the manufacturer's protocol via a flow cytometer.

### In Vitro Macrophage Polarization Analysis in RAW264.7 Cells

LPS was applied to induce macrophage polarization, while CD80 and CD206 were chosen to mark the M1 and M2 phenotypes, respectively. APC‐conjugated anti‐CD80 (104 714, Biolegend) and FITC‐conjugated anti‐CD206 (141 703, Biolegend) were used to evaluate macrophage subsets. RAW264.7 macrophages were planted in a six‐well plate at a density of 1 × 10^6^ cells per well overnight to allow attachment, then the cells were preincubated with MTem/Los (Tem: 600 ng mL^−1^, Los: 50 µg mL^−1^ at a final concentration) for 1 h and then stimulated with LPS (1 µg mL^−1^) for 24 h. After collection, samples were treated according to the manufacturer's protocol and further analyzed via a flow cytometer.

### Dry Eye Model Mice

All animal experiments have complied with the Association for Research in Vision and Ophthalmology Statement for the Use of Animals in Ophthalmic and Vision Research and the guidelines for Animal Care and Use Committee, Zhejiang University. All animal experiments were approved by the Animal Ethics Committee, the Second Affiliated Hospital, School of Medicine, Zhejiang University (Approval number: 2020–40). Sixty C57BL/6 mice (female, 8 weeks, 20 ± 2 g) were provided by SLAC Laboratory Animal Co., Ltd. The mice were housed in a standard environment with constant temperature (22 ± 1 °C) and a regular light/dark (12 h/12 h) cycle with ad libitum access to food and water. BAK was used to induce experimental DED in mice. Briefly, each right eye of the mice was administered with 5 µL of 0.2% BAK eye drops (w/v) twice per day for 14 consecutive days. Then, the mice were randomly divided into six groups and received topical instillation of 10 µL of 0.9% saline (w/v), Los (15.6 µg kg^−1^), Tem (2.5 mg kg^−1^), MTem (Tem: 2.5 mg kg^−1^), MTem/Los (Tem: 2.5 mg kg^−1^, Los: 15.6 µg kg^−1^), and Restasis (Allergan) twice per day, respectively.

### Precorneal Retention Evaluation

In vivo imaging under anesthesia and cryopreserved sections of mice eyeballs without anesthesia were conducted to assess precorneal retention time. For in vivo imaging detection, the dry eye model mice were anesthetized via peritoneal administration of 300 mg kg^−1^ sodium pentobarbital and instilled with 5 µL of MTem‐Rho (rhodamine B concentration: 10 mm equivalent to that of free rhodamine B) and free rhodamine B (10 mm) onto the right eyes. Later, the variation of fluorescence intensity of the mouse eye was recorded using the in vivo imaging system (Lumina LT, PerkinElmer) at distinct time points (0, 0.5, 3, 5, 15, 25 min) (Ex/Em, 535 nm/600 nm). For cryopreserved section imaging, the same eye drops of MTem‐Rho and Rho as aforementioned were added to the corneal surfaces of dry eye model mice under consciousness. Then, mice were executed at the scheduled time as 2 and 10 min precisely after administration, soon after which their eyeballs were carefully taken out from orbits and frozen in dry ice with optimum cutting temperature (OCT) (4583, SAKURA) incubated. The 7 µm frozen slices were freshly sectioned and then observed under a fluorescence microscope. The image intensity was calculated by ImageJ software.

### Therapeutic Efficacy Assessment

All mice were anesthetized via peritoneal administration of 300 mg kg^−1^ sodium pentobarbital. Clinical assessments for dry eye, including tear volume conducted as Schirmer Test, tear break‐up time (TBUT), and ocular surface parameters mainly measured by sodium fluorescein staining, were utilized to test the therapeutic efficacy. Tear volume was measured using phenol red‐impregnated cotton threads (Jing Ming Tech Co., Ltd). The cotton thread was placed on the lower eyelid palpebral conjunctiva at 1/3 of the distance from the lateral canthus for 15 s. The length of the wetted cotton thread was determined in millimeters using a vernier caliper. TBUT was recorded in seconds by pausing a stopwatch when the first black spot was observed using a slit lamp image system (YZ5T, 66 Vision‐Tech Co., Ltd) under cobalt blue light. Ten right eyes per group were measured 3 times, and the average value was calculated for analysis. As for ocular fluorescein staining, 2 µL of 1% sodium fluorescein (w/v) were instilled into the inferior conjunctival sac and eyelids, and subsequently, the eyelids were manually closed 3–4 times. The fluorescein staining images were captured under the cobalt blue light by a digital slit lamp, and the fluorescein corresponding scores were graded in accordance with a previous report. Briefly, a grade of 0–4 was assigned to each quadrant: 0, absent; 1) slightly punctate staining <30 spots; 2) punctate staining > 30 spots, but not diffuse; 3) severe diffuse staining but no positive plaque; and 4) positive fluorescein plaque‐staining score. Ten right eyes per group were examined and the average value was used for analysis.

### Safety Evaluation

Fifty mice, randomly divided into five groups, received a topical instillation on the right eye of 10 µL of 0.9% saline (w/v), Los (15.6 µg kg^−1^), Tem (2.5 mg kg^−1^), MTem (Tem: 2.5 mg kg^−1^), MTem/Los (Tem: 2.5 mg kg^−1^, Los: 15.6 µg kg^−1^) twice per day, respectively, for 14 consecutive days. Their body weights were measured at a fixed time (8 AM) at 0, 4, 9, and 14 days of administration. On the 14th day, the right eyes of the mice were examined under a digital slit lamp and stained by fluorescein sodium under bright and cobalt blue light. After a sacrifice by overdose anesthesia, the right eyeballs of each mouse were carefully taken out and made into H&E sections. Besides, on the 30th day after treatment, the significant organs, including liver, lung, heart, kidney, and spleen, were quickly extracted once the mice were executed, followed by being manufactured as H&E staining specimens to further investigate in vivo biosafety.

### Statistical Analyses

The results were expressed as the mean ± standard deviation (SD). All statistical analyses were performed using the GraphPad Prism software. An independent‐sample *t*‐test was used to compare between two groups. For the comparison among (more than) three groups, comparative studies of means were carried out using one‐way ANOVA analysis. The difference was represented as no significant difference as ns where *p* > 0.05, statistically significant where *p < 0.05, and very significant where **p < 0.01.

## Conflict of Interest

The authors declare no conflict of interest.

## Supporting information

Supporting Information

## Data Availability

The data that support the findings of this study are available in the supplementary material of this article.
