# Supplementary material for: Anti‐Oxidative and Anti‐Inflammatory Micelles: Break the Dry Eye Vicious Cycle
Source: Adv Sci (Weinh). 2022 Apr 18;9(17):2200435. doi: 10.1002/advs.202200435 (PMC9189644; doi:10.1002/advs.202200435)
Supplement: Supplementary file 1 — Supporting Information [file ADVS-9-2200435-s001.pdf]

ADVANCED SCIENCE
Open Access

Supporting Information

for *Adv. Sci.*, DOI 10.1002/advs.202200435

Anti-Oxidative and Anti-Inflammatory Micelles: Break the Dry Eye Vicious Cycle

Su Li, Zhouyu Lu, Yue Huang, Yin Wang, Qiao Jin, Xingchao Shentu, Juan Ye, Jian Ji, Ke Yao* and Haijie Han**

ADVANCED SCIENCE
Open Access

Supporting Information

for *Adv. Sci.*, DOI 10.1002/advs.202200435

Anti-Oxidative and Anti-Inflammatory Micelles: Break the Dry Eye Vicious Cycle

Su Li, Zhouyu Lu, Yue Huang, Yin Wang, Qiao Jin, Xingchao Shentu, Juan Ye, Jian Ji, Ke Yao* and Haijie Han**

Supporting Information

Anti-oxidative and Anti-inflammatory Micelles: Break the Dry Eye Vicious Cycle

Su Li, Zhouyu Lu, Yue Huang, Yin Wang, Qiao Jin, Xingchao Shentu, Juan Ye, Jian Ji, Ke Yao*, and Haijie Han**

Experimental Section

Materials: N6-Carbobenzoxy-l-lysine N-carboxyanhydride (Lys(CBz)-NCA), anhydrous N,N'-dimethylformamide (DMF), trifluoroacetic acid (TFA), hydrobromic acid solution (33 wt% in acetic acid), 1-(3-Dimethylaminopropyl)-3-ethylcarbodiimide hydrochloride (EDC), and N-Hydroxysuccinimide (NHS) were provided by J&K Scientific Ltd. l-phenylalanine N-carboxyanhydride (Phe-NCA) was received from Ark Pharm, Inc. Methoxypolyethylene glycol amine (Mw: 2000, mPEG-NH_2) was obtained from Seebio Biotech Co., Ltd. Benzalkonium chloride (BAK), 4-Carboxy-2,2,6,6-tetramethylpiperidine 1-Oxyl (4-carboxy-TEMPO, denoted as Tem), and lysozyme from hen egg white was purchased from Sigma-Aldrich. Diphenylhydrazine was received from Accustandard Inc. Losmapimod (Los) was provided by Kailiqi Biopharma Technology Co., Ltd. MilliQ Water 18.2 (MΩ cm^{-1}) was obtained using a Millipore MilliQ Academic Water Purification System. Other reagents were used as received unless specifically noted.

Synthesis of Cationic Amphiphilic Polypeptide (PEG-b-(Lys-co-Phe)): The cationic amphiphilic polypeptide PEG-*b*-(Lys-*co*-Phe) was synthesized by α-amino acid *N*-carboxyanhydride (NCA) ring-opening copolymerization of Lys(CBz)-NCA and Phe-NCA monomers using mPEG-NH_2 as the initiator, followed by de-protection of the CBz group. In detail, 200 mg of mPEG-NH_2 (0.2 mmol), 1837 mg of Lys(CBz)-NCA (6.0 mmol), 765 mg of Phe-NCA (4.0 mmol), and 25 mL of anhydrous DMF were added into a 100 mL flask under a nitrogen atmosphere. The mixture solution was then degassed by three freeze-pump-thaw cycles and stirred at 30 °C. After 72 h, the crude product was precipitated using 200 mL of HCl (1 M) and washed with water 3 times to remove excess DMF. White solid products PEG-*b*-

(Lys(CBz)-*co*-Phe) were obtained under a vacuum. Then, 100 mg of PEG-*b*-(Lys(CBz)-*co*-Phe) was dissolved in 2 mL of TFA in an ice-water bath. 200 mL of hydrobromic acid solution (33 wt% in acetic acid) (33% w/w) was added dropwise, and the mixture was continuously stirred for 2 h to de-protect the CBz groups. The reaction mixture was precipitated directly into diethyl ether 3 times. White solid products PEG-*b*-(Lys-*co*-Phe) were obtained under vacuum.

Synthesis of Cationic Amphiphilic Anti-oxidative Polypeptide (PEG-b-(Lys-graft-Tem-co-Phe)): The cationic amphiphilic anti-oxidative polypeptide (PEG-*b*-(Lys-*graft*-Tem-*co*-Phe)) was synthesized by amide reaction of the amino groups in PEG-*b*-(Lys-*co*-Phe) with the carboxyl groups in Tem. In detail, 120 mg of Tem (0.6 mmol), 192 mg of EDC (1 mmol), and 116 mg of NHS (1 mmol) were added into 16mL of DMSO and stirred for 1 h at room temperature. Then 134 mg of PEG-*b*-(Lys-*co*-Phe) in 4 mL of DMSO was added and continuously stirred for 24 h at room temperature. Finally, the reaction mixture was extensively dialyzed against DMSO (molecular weight cutoff (MWCO) 1000) and then distilled water (MWCO 1000). Faintly yellow products (PEG-*b*-(Lys-*graft*-Tem-*co*-Phe)) were obtained through lyophilization.

Characterizations: Proton nuclear magnetic resonance (^{1}H-NMR) spectra were conducted on a Bruker DMX500 (Bruker). Electron paramagnetic resonance (EPR) spectra were obtained using a Bruker EPR A300 (Bruker) equipped with an ER-4119HS resonator. The magnetic field was calibrated by an NMR probe and the cavity frequency was ~9.85 GHz. Samples in DMSO solution were placed in quartz tubes and their EPR spectra were obtained with a modulation amplitude of 2.0 G at a frequency of 100 kHz. Other measurement parameters include centerfield, 3514.7 G; sweep width, 120 G; microwave power, 20.39 mW; and sweep time, 20.00 s. Dynamic light scattering (DLS) measurements were carried out on a Zetasizer Nano-ZS (Malvern Instruments) equipped with a He-Ne laser at a wavelength of 633 nm at 25 °C. The intensity-average hydrodynamic diameter (D_h) was adopted in this research. The samples were cleaned using a 0.45 μm Millipore filter before the measurements. Transmission electron microscopy (TEM) was recorded on an HT7700 TEM (HITACHI) operated at 100 kV. UV-vis spectra were performed using an Evolution 300 UV-visible spectrophotometer (Thermo Scientific) and the absorption was recorded from 200 to 400 nm. The released Los solution was passed through a 0.22 μm Millipore filter and measured by high-performance liquid

chromatography (HPLC) using Agilent 1200 (Agilent). The mobile phase consisted of acetonitrile and H_2O (30/70, v/v) with a flow rate of 1.5 mL min^{-1}.

Cell Lines and Cell Cultures: Human corneal epithelial cell lines (HCECs) were purchased from American Type Culture Collection (ATCC). HCECs were incubated in 25 cm^2 cell culture flasks (Corning) and cultured in DMEM/F12 (Gibco), containing 10% fetal bovine serum (Ausgenex), penicillin (100 U mL^{-1}), and streptomycin (100 μg mL^{-1}) (Gibco) at 37 °C in a humidified 5% CO_2 atmosphere. The cells were passaged with 0.25% trypsin containing 0.02% ethylene diamine tetra-acetic acid (EDTA) (Gibco) every 3 days. RAW264.7 macrophages were obtained from ATCC and raised in 10 cm^2 dishes, cultured in DMEM medium containing 4.5 g L^{-1} D-Glucose (Corning) supplemented with 10% fetal bovine serum (Ausgenex) plus penicillin (100 U mL^{-1}) and streptomycin (100 μg mL^{-1}) (Gibco). The macrophages were passed every 2 days with cell scrapers.

RNA Isolation, cDNA Synthesis, and Quantitative PCR (Q-PCR) Analysis: The total RNAs collected from the HCECs and RAW264.7 cells were extracted using TRIzol Reagent (15596018, Invitrogen) according to the manufacturer's protocol. RNA quantitation was recorded using a spectrophotometer (NanoDrop 2000c, Thermo Scientific). Qualified RNA samples (260/280 ratio > 1.8, 260/230 ratio ≥ 1.0, concentration ≥ 100 ng μL^{-1}) were used for reverse transcription. Isolated RNA was reverse transcribed into cDNA using the Primescript RT Master Mix (RR036A, TaKaRa) with incubation at 37 °C for 15 min and, then, at 85 °C for 5 s, subsequently terminated by incubation at 4 °C forever using a commercial Thermal Cycler (C1000, Bio-Rad). Q-PCR analysis was performed by TB Green® Premix Ex Taq™ II reagents (RR820A, TaKaRa) and conducted using a 7500 Fast Real-Time PCR System (Applied Biosystems): Holding Stage: 95 °C for 30 s. Cycling Stage: 40 cycles of 95 °C for 3 seconds, 60 °C for 30 s. Quantitative analysis was performed using the comparative (ΔΔCT) method and Each assay was normalized by amplifying the housekeeping cDNA h-GAPDH and m-actin. Table S1 lists tested cytokines and sequences of forward and reverse primers.

Table S1. Primers used in quantitative RT-PC

Target	Forward Primer	Reverse Primer
h-GAPDH	GTCTCCTCTGACTTCAACAGCG	ACCACCCTGTTGCTGTAGCCAA
h-MMP-9	GAACCAATCTCACCGACAGG	GCCACCCGAGTGTAACCATA
h-IL-1β	CCACAGACCTTCCAGGAGAATG	GTGCAGTTCAGTGATCGTACAGG
m-actin	TTCGTTGCCGGTCCACACCC	GCTTTGCACATGCCGGAGCC
m-MMP-9	GCAGAGGCATACTTGTACCG	TGATGTTATGATGGTCCCACTTG
m-IL-1β	AAAAAAGCCTCGTGCTGTCG	GTCGTTGCTTGGTTCTCCTTG

Western Blotting Analysis: Proteins were extracted by using RIPA Lysis Buffer I Kit (C500005, Sangon) containing 1 mM phenylmethanesulfonyl fluoride (PMSF), protease inhibitor cocktail (1 μM E-64, 0.8 μM aprotinin, and 1 μM leupeptin), and phosphatase inhibitor cocktail (10 mM sodium fluoride, 1 mM sodium orthovanadate, 25 mM sodium β-glycerophosphate, and 1 mM sodium pyrophosphate) on ice, followed by being centrifuged at 1.2×10^4 g for 15 min at 4 °C. The total protein concentration was determined with a BCA protein assay kit (23227, Thermo Scientific). Equal amounts of protein (20-40 μg) were then loaded on 10% tris-glycine SDS polyacrylamide gels and electronically transferred to polyvinylidene difluoride (PVDF) membranes (IPVH00010, Immobilon). After being blocked with 5% BSA for 1 h, the membranes were then subjected to primary antibodies overnight at 4 °C and secondary antibodies at room temperature for 1 h. The specific protein bands were visualized by a hypersensitive ECL Chemiluminescent Substrate (BL520a, Biosharp) and imaged using a commercial imaging system (ChemiDoc MP, Bio-Rad). The image intensity was calculated by the ImageJ software. GAPDH was served as an internal control. All antibodies are listed in Table S2.

Table S2. Antibodies for Western Blotting and Immunofluorescent Staining

Antibody	Company	Category number	Examination Type	Dilution
Primary antibody				
rabbit anti-MMP-9	Proteintech	10375-2-AP	Western Blotting	1:500
rabbit anti-IL-1β	Abcam	ab9722	Western Blotting	1:4000
rabbit anti-p38 MAPK	CST	9218t	Western Blotting	1:1000
rabbit anti-p-p38 MAPK	CST	4511T	Western Blotting	1:1000
NF-κB p-p65	CST	3033T	Western Blotting	1:1000
rabbit anti-GAPDH	CST	8884	Western Blotting	1:2000
rabbit anti-MMP-9	Proteintech	10375-2-AP	Immunofluorescent Staining	1:200
rabbit anti-IL-1β	Abcam	ab9722	Immunofluorescent Staining	1:400
rabbit anti-Ki67	Abcam	ab243878	Immunofluorescent Staining	1:400
rabbit anti-K10	Abcam	ab76318	Immunofluorescent Staining	1:400
Secondary antibody				
HRP-conjugated anti-rabbit IgG	CST	7074	Western Blotting	1:5000
Goat anti-rabbit IgG (H+L), Alexa Fluor 555	Invitrogen	A-21428	Immunofluorescent Staining	1:1000

Transcriptome Analysis of HS-induced HCECs and LPS-induced RAW264.7 Cells: HCECs incubated with normal medium (HCEC group, n = 3), hyperosmotic medium (500 mOsm L^{-1})

(HS group, n = 3), and hyperosmotic medium pretreated with MTem/Los (Tem: 600 ng mL^{-1}, Los: 50 μg mL^{-1} at a final concentration) for 1 h (HS + MTem/Los group, n = 3) groups were collected for RNA-sequencing. As for RAW264.7 macrophages, cells incubated with normal medium (RAW264.7 group, n = 3), LPS (1μg mL^{-1}) (LPS group, n = 3), and LPS pretreated with MTem/Los (Tem: 600 ng mL^{-1}, Los: 50 μg mL^{-1} at a final concentration) for 1 h (LPS + MTem/Los group, n = 3) groups were harvested for RNA-sequencing.

RNA-sequencing: Total RNA of HCECs and RAW264.7 cells of each sample were extracted as recorded above in the RNA isolation method. RNA integrity and concentration were examined utilizing an Agilent 2100 Bioanalyzer (Agilent Technologies, Inc., Santa Clara, CA, USA). The mRNA was isolated by NEBNext Poly (A) mRNA Magnetic Isolation Module (NEB, E7490). The cDNA library was constructed following the manufacturer's instructions of NEBNext Ultra RNA Library Prep Kit for Illumina (NEB, E7530) and NEBNext Multiplex Oligos for Illumina (NEB, E7500). In brief, the enriched mRNA was fragmented into approximately 200nt RNA inserts, which were used to synthesize the first-strand cDNA and the second cDNA. The double-stranded cDNA was performed end-repair/dA-tail and adaptor ligation. The suitable fragments were isolated by Agencourt AMPure XP beads (Beckman Coulter, Inc.), and enriched by PCR amplification. Finally, the constructed cDNA libraries of HCECs and RAW264.7 cells were sequenced on a flow cell using an Illumina HiSeq™ sequencing platform.

Mice Sacrifice and Specimen Disposal: After different treatments of Saline, Los, Tem, MTem, MTem/Los, and Restasis® instillations for 4 days as described previously, all experimental mice were sacrificed through overdose anesthesia. The right eyeballs accompanying peri-conjunctiva tissues of mice were gently dissected. Six ocular specimens in each group were fixed in 4% formalin for 48 h, dehydrated, and, then embedded in paraffin. Four other ocular specimens per group were excised and then quickly embedded in the OCT compound. OCT-incubated specimens were sectioned into 7 μm vertical slices as cryopreserved sections using a slider (CM1950, LEICA) and then stored at -80 °C.

Histological Assessment: Paraffin-embedded specimens were sectioned into 4 μm vertical slices using a slider (RM2245, LEICA) and subsequently subjected to hematoxylin and eosin (H&E) staining. Corneal tissue histological analysis was conducted under a digital light

microscope (DM2500, LEICA). Periodic Acid-Schiff stain kit (G1280, Solarbio) was used for periodic acid-Schiff (PAS) staining on 4 μm thick dewaxed paraffin sections according to the manufacturer's protocol. Representative images of the conjunctival sections were captured with a digital light microscope. The thickness and areas of positively-stained conjunctival goblet cells were counted from five samples per group by using ImageJ. Results were expressed as a percentage of the Saline group.

ROS Assessment: Cryopreserved sections were washed 3 times with PBS and then incubated with Dihydroethidium (D7008, Sigma) at 37 °C in the dark for 30 minutes, followed by being counterstained with DAPI Fluoromount-GTM for 20 minutes, after which the sections were observed and captured with a fluorescence microscope. The average ROS fluorescence intensities were determined by ImageJ.

Immunofluorescence Staining: Cryopreserved sections were washed with PBS for 20 minutes, incubated with 0.5% TritonX-100 for 20 min, and then blocked with 10% Normal Goat Serum (AR1009, Boster) at room temperature for 60 min. The sections were incubated with the primary antibodies at 4 °C overnight. After 3 washes in PBS, the next day, the samples were subsequently incubated with the second antibody for 1 h at room temperature and counterstained with DAPI Fluoromount-GTM for 20 min. The staining was observed and captured with a fluorescence microscope. The average positive fluorescence intensities from three samples per group were determined by ImageJ. Details of all antibodies are listed in Table S2.

TdT-mediated dUTP Nick-end Labeling (TUNEL) Staining: TUNEL Staining on mice eye cryosections was conducted using an In Situ Cell Death Detection Kit, Fluorescein (11684795910, Roche) according to the manufacturer's protocol. Sections were counterstained with DAPI Fluoromount-GTM (36308ES11, Yeasen) for 20 minutes and the representative images of TUNEL-positive cells on the cornea were captured with a fluorescence microscope. The average TUNEL fluorescence intensities were determined by ImageJ.

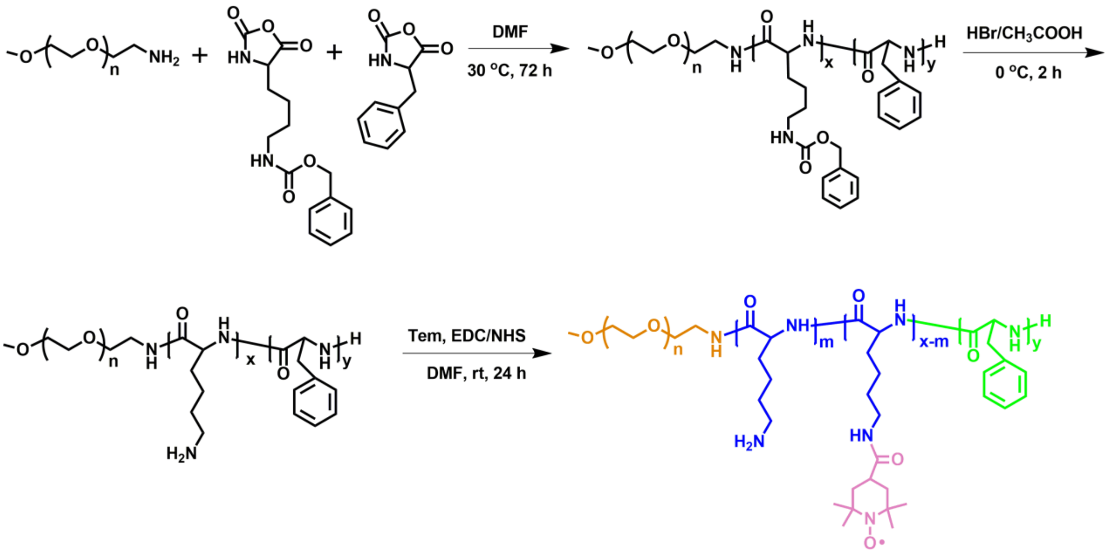

Figure S1. Synthetic routes of PEG-*b*-(Lys-*graft*-Tem-*co*-Phe).

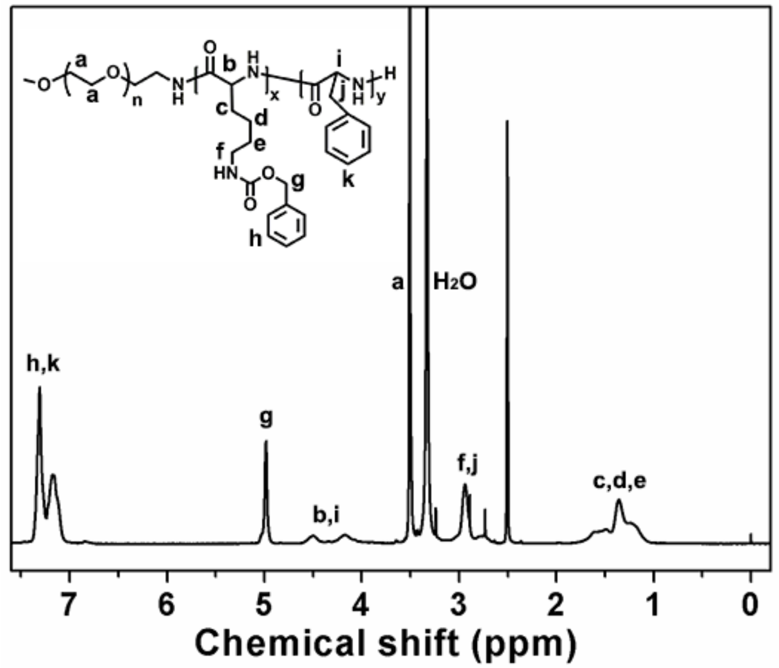

Figure S2. ^{1}H-NMR spectrum of PEG-*b*-(Lys (CBz)-*co*-Phe) with DMSO-d_6 as reference solvent.

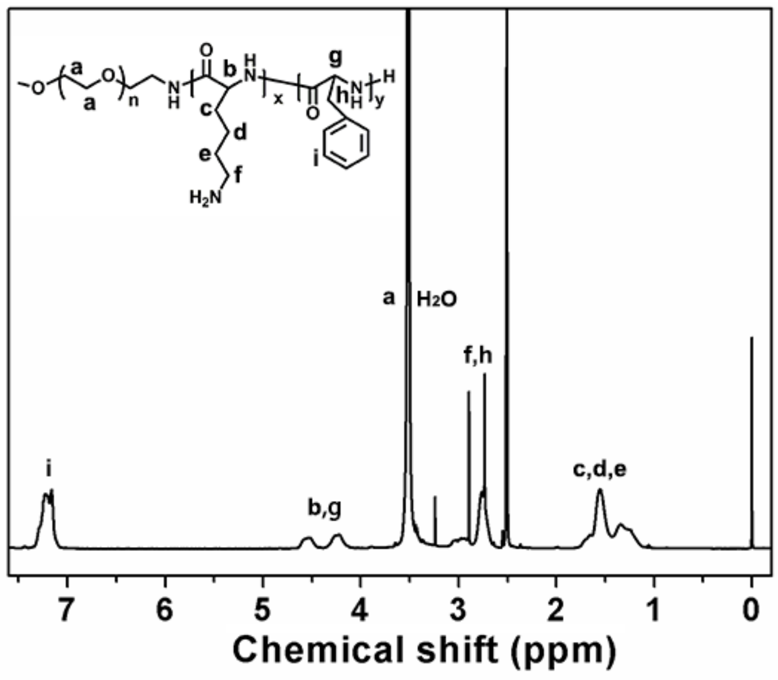

Figure S3. The ^{1}H-NMR spectra of PEG-*b*-(Lys-*co*-Phe) with DMSO-d_6 as the reference solvent.

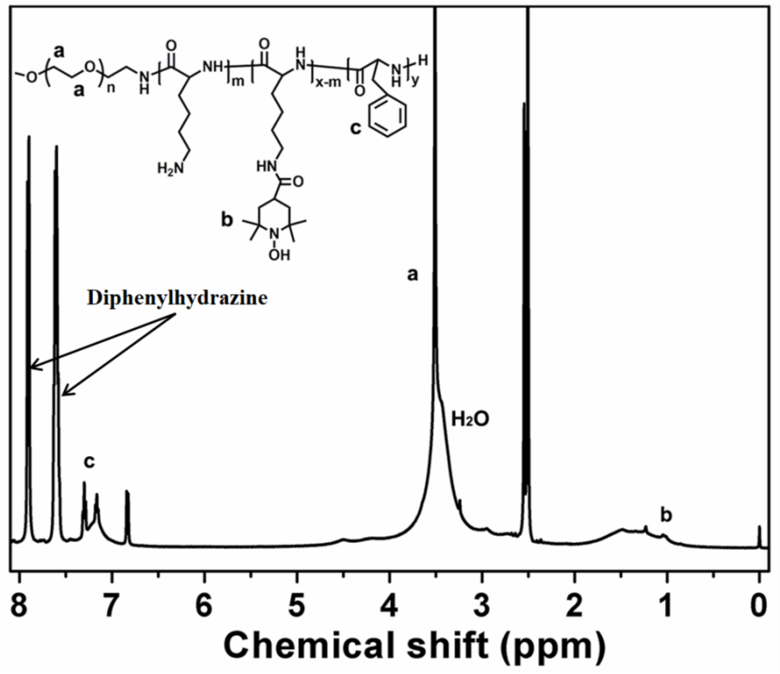

Figure S4. The ^{1}H-NMR spectra of PEG-*b*-(Lys-*graft*-Tem-*co*-Phe) with DMSO-d_6 as the reference solvent.

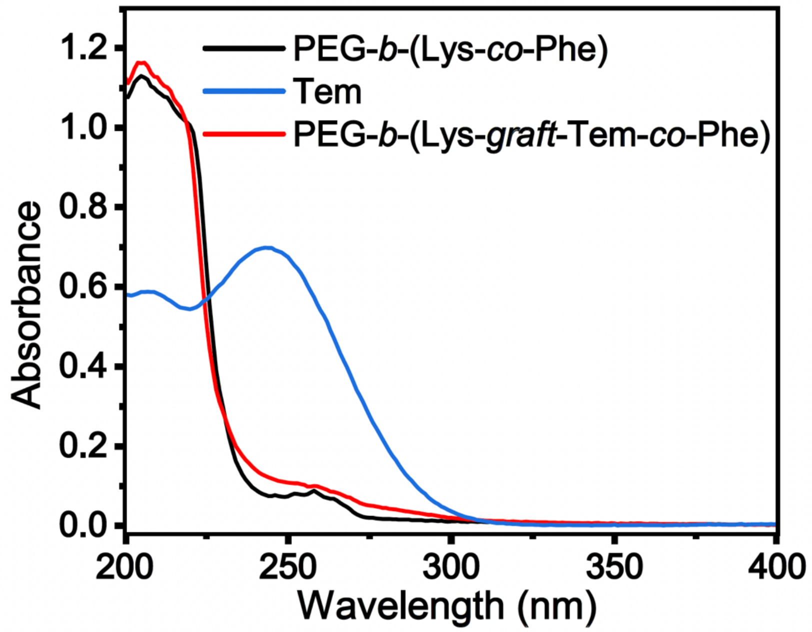

Figure S5. UV-vis spectra of PEG-*b*-(Lys-*co*-Phe), Tem, and PEG-*b*-(Lys-*graft*-Tem-*co*-Phe).

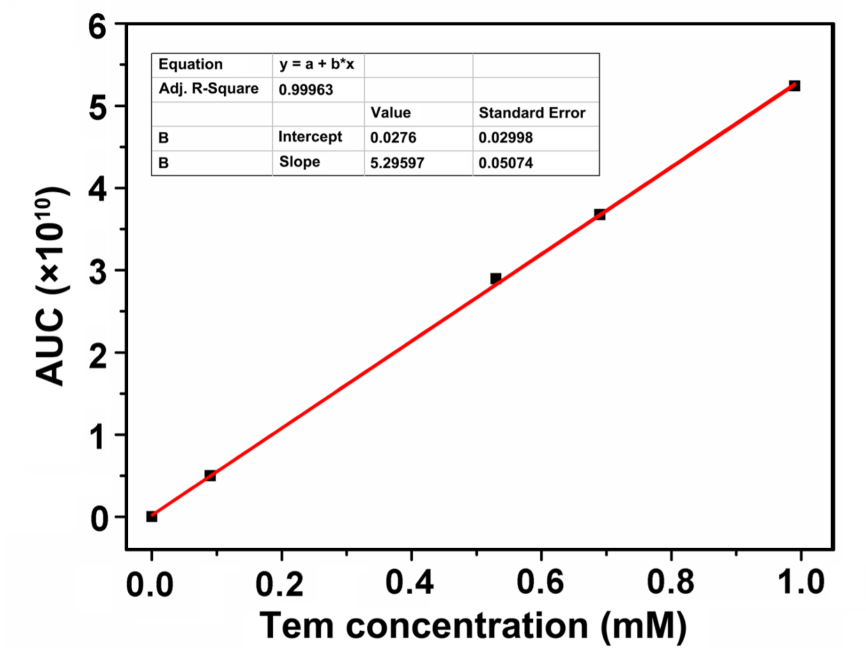

Figure S6. The calibration curve of Tem by EPR.

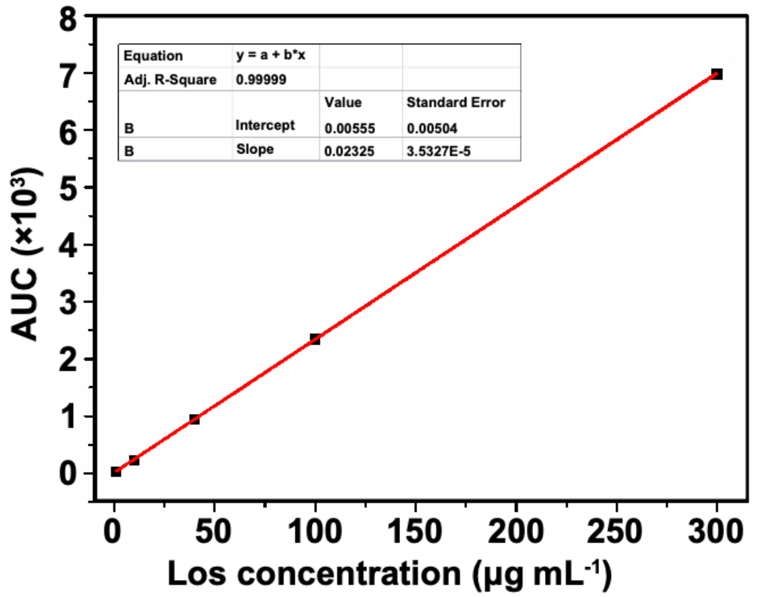

Figure S7. The calibration curve of Los by HPLC.

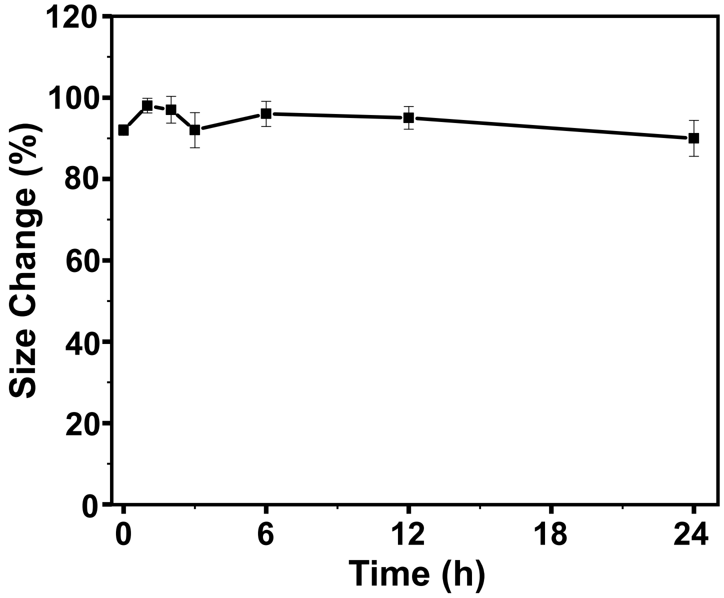

Figure S8. The size change of MTem/Los after adding lysozyme in PBS.

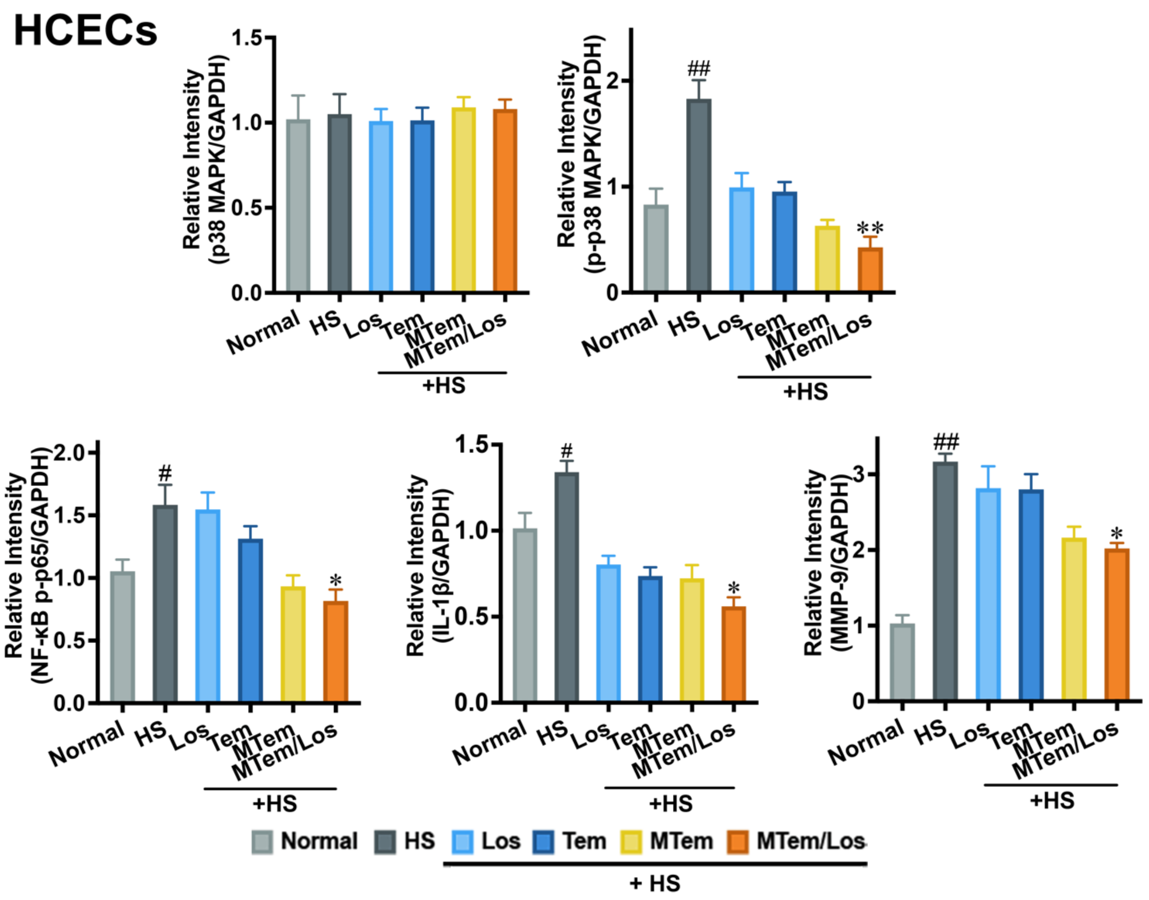

Figure S9. Quantitative analysis of p38 MAPK, p-p38 MAPK, NF-κB p-p65, IL-1β, and MMP-9 protein levels in hyperosmotic stress (HS) (500 mOsm L^{-1}) induced HCECs. Data were presented as mean ± SD; n = 3. $^{\#}p < 0.05$ *vs.* the Normal group, $^{\#\#}p < 0.01$ *vs.* the Normal group, $^{*}p < 0.05$ *vs.* the HS group, and $^{**}p < 0.01$ *vs.* the HS group.

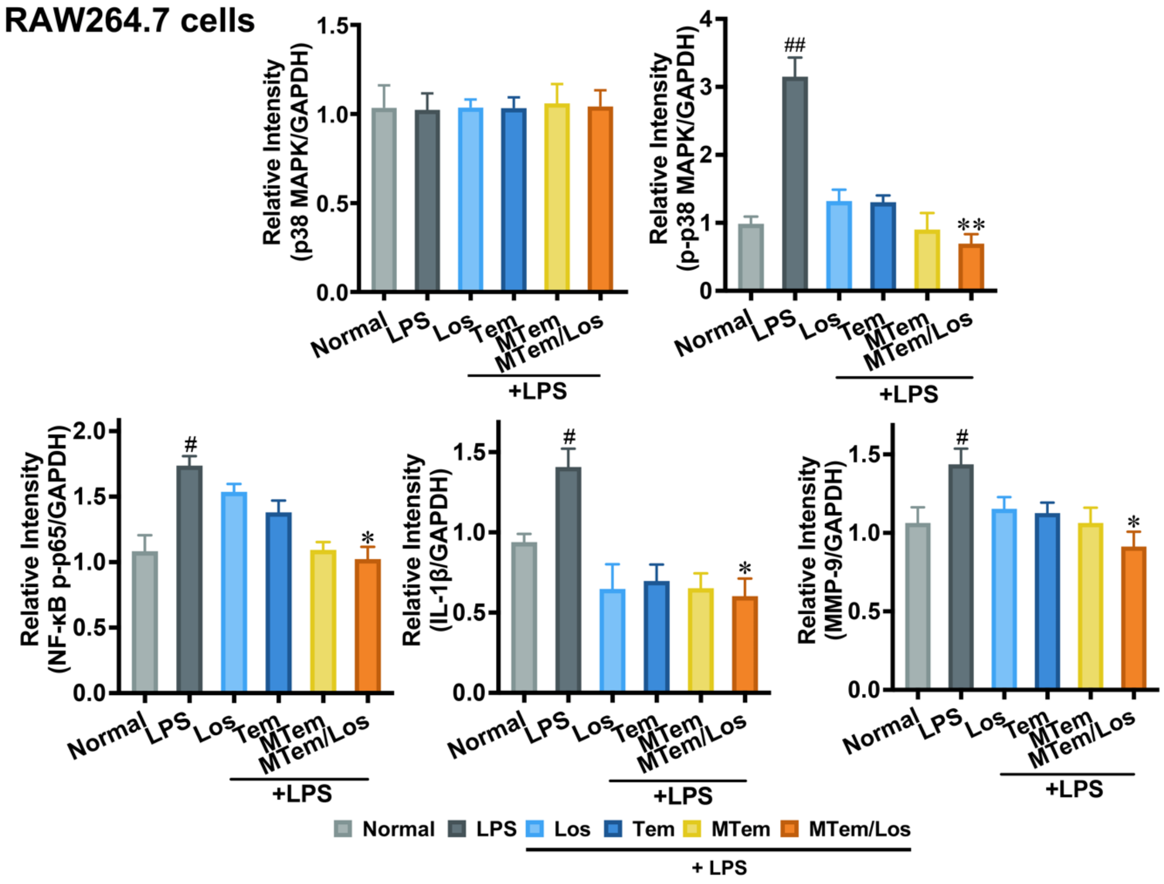

Figure S10. Quantitative analysis of p38 MAPK, p-p38 MAPK, NF-κB p-p65, IL-1β, and MMP-9 protein levels in LPS induced RAW264.7 cells. Data were presented as mean ± SD; n = 3. $^{\#}p < 0.05$ *vs.* the normal group, $^{\#\#}p < 0.01$ *vs.* the normal group, $^{*}p < 0.05$ *vs.* the LPS group, and $^{**}p < 0.01$ *vs.* the LPS group.

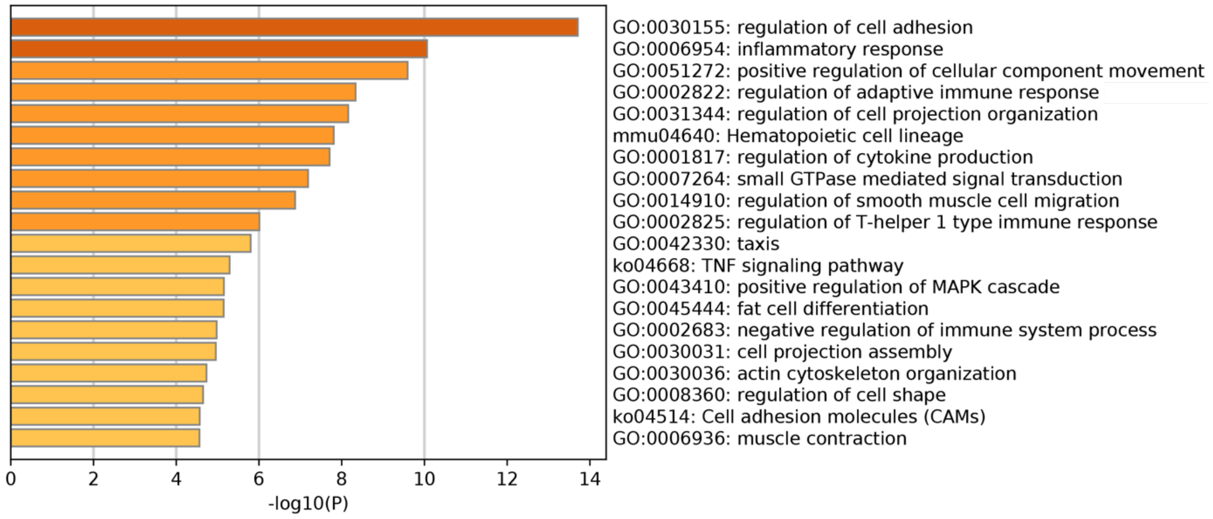

Figure S11. KEGG pathway enrichment analysis of DEAS with enrichment scores in HCECs. Main enriched pathways with significance were shown.

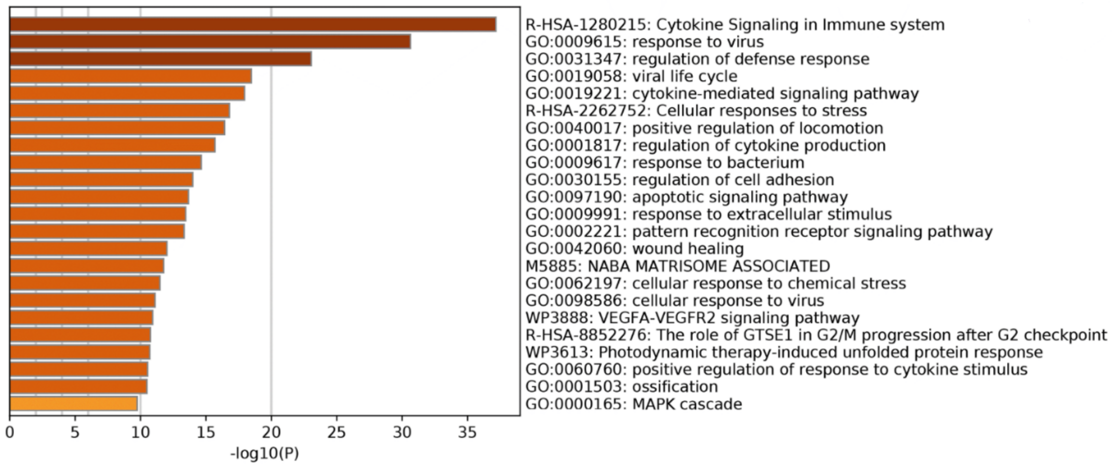

Figure S12. KEGG pathway enrichment analysis of DEAS with enrichment scores in RAW264.7 cells. Main enriched pathways with significance were shown.

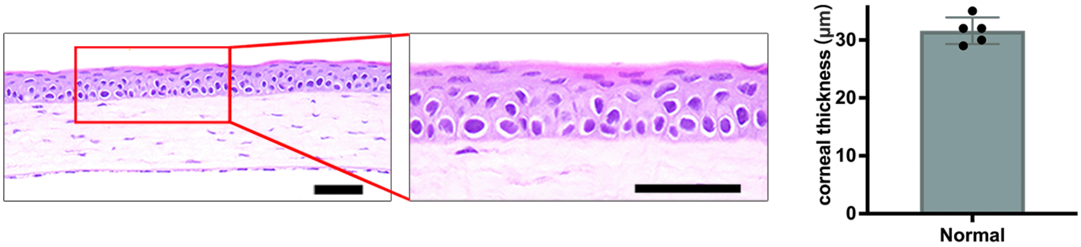

Figure S13. Representative H&E staining images of the cornea and corresponding amplifying corneal epithelium of normal mouse. The scale bars are 50 μm.

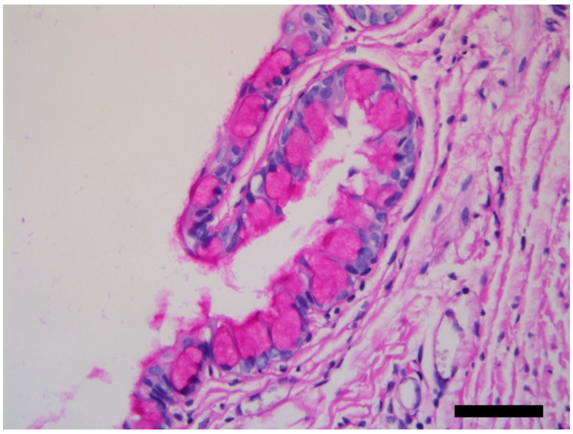

Figure S14. Representative conjunctival PAS staining image of the normal mouse. The scale bar is 50 μm.

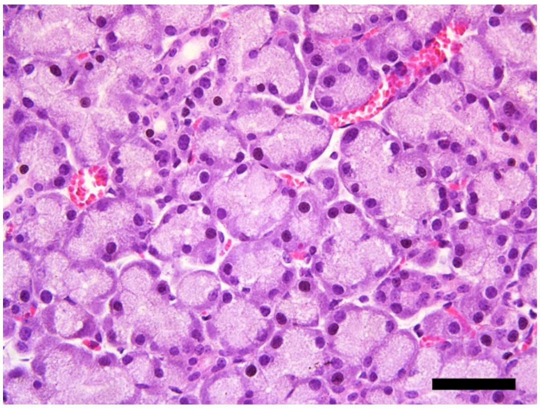

Figure S15. Representative lacrimal gland H&E staining image of the normal mouse. The scale bar is 50 μm.

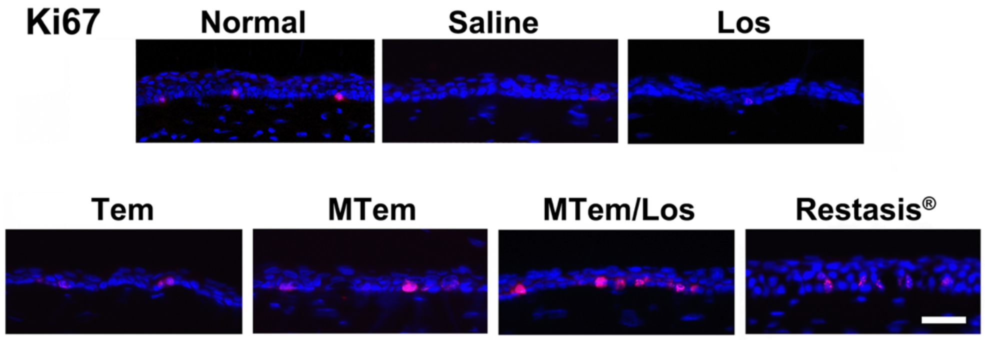

Figure S16. Representative features of proliferation (Ki67) immunostaining on the corneal epithelial cells in the mice eyes after 14 days of diverse therapies for DED. The scale bar is 50 μm.

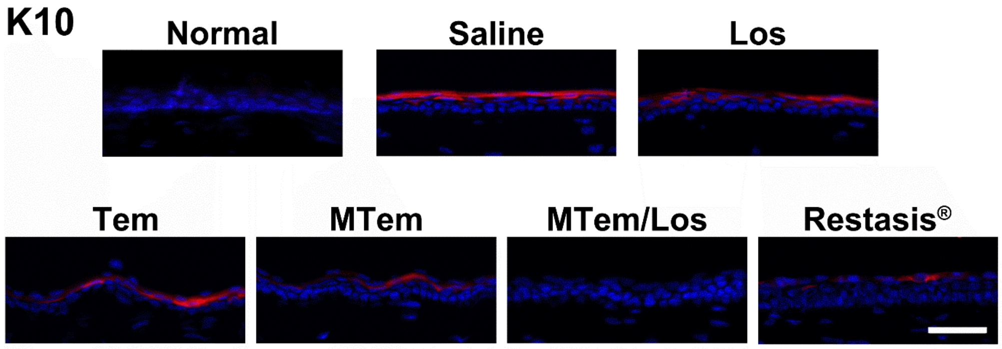

Figure S17. Representative features of keratinization (K10) immunostaining on the corneal epithelial cells in the mice eyes after 14 days of diverse therapies for DED. The scale bar is 50 μm.

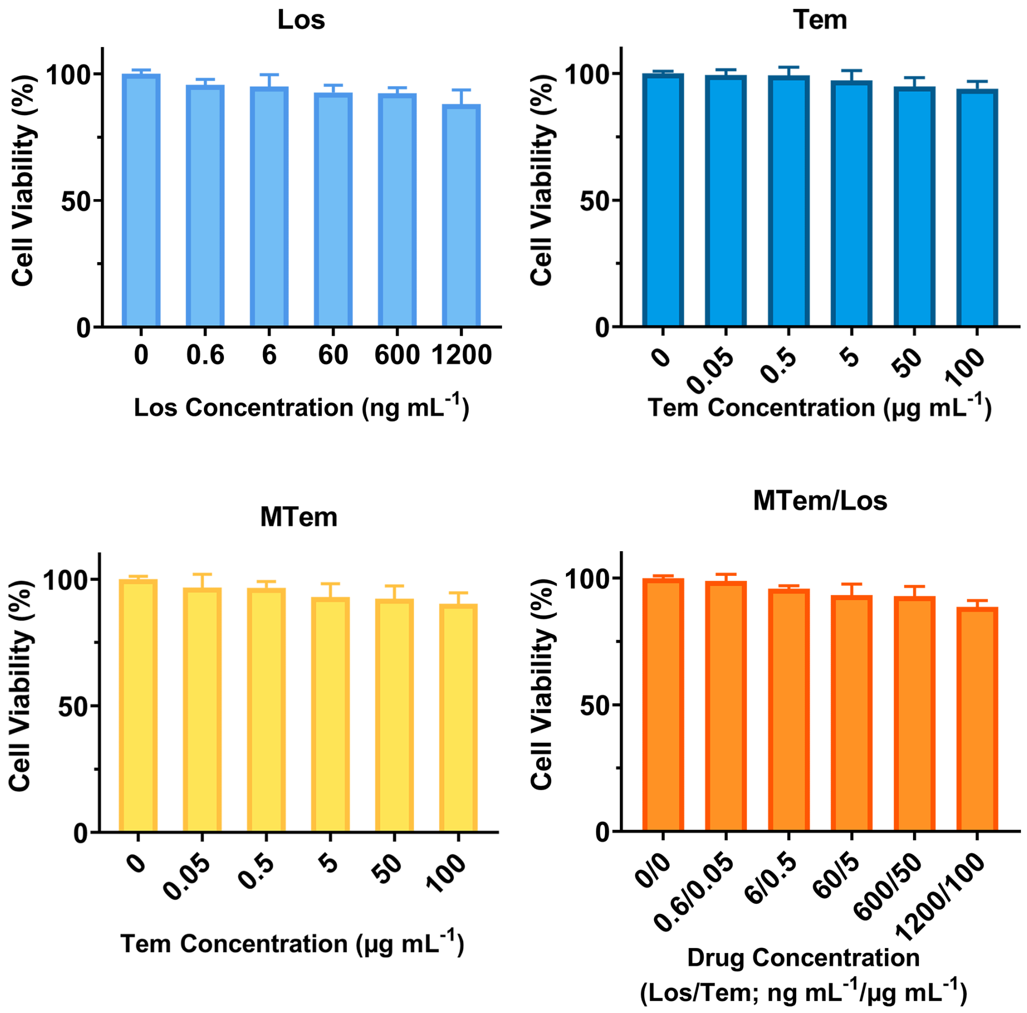

Figure S18. Effect of Los, Tem, MTem, and MTem/Los in the cultured HCECs viability by CCK-8 assay. HCECs were treated with Los, Tem, MTem and MTem/Los (with a final concentration of Los/Tem: 0 ng mL^{-1}/0 μg mL^{-1}, 0.6 ng mL^{-1}/0.05 μg mL^{-1}, 6 ng mL^{-1}/0.5 μg mL^{-1}, 60 ng mL^{-1}/5 μg mL^{-1}, 600 ng mL^{-1}/50 μg mL^{-1}, and 1200 ng mL^{-1}/100 μg mL^{-1}) for 24 h, followed by CCK-8 assay. Data were presented as mean ± SD; n = 5.

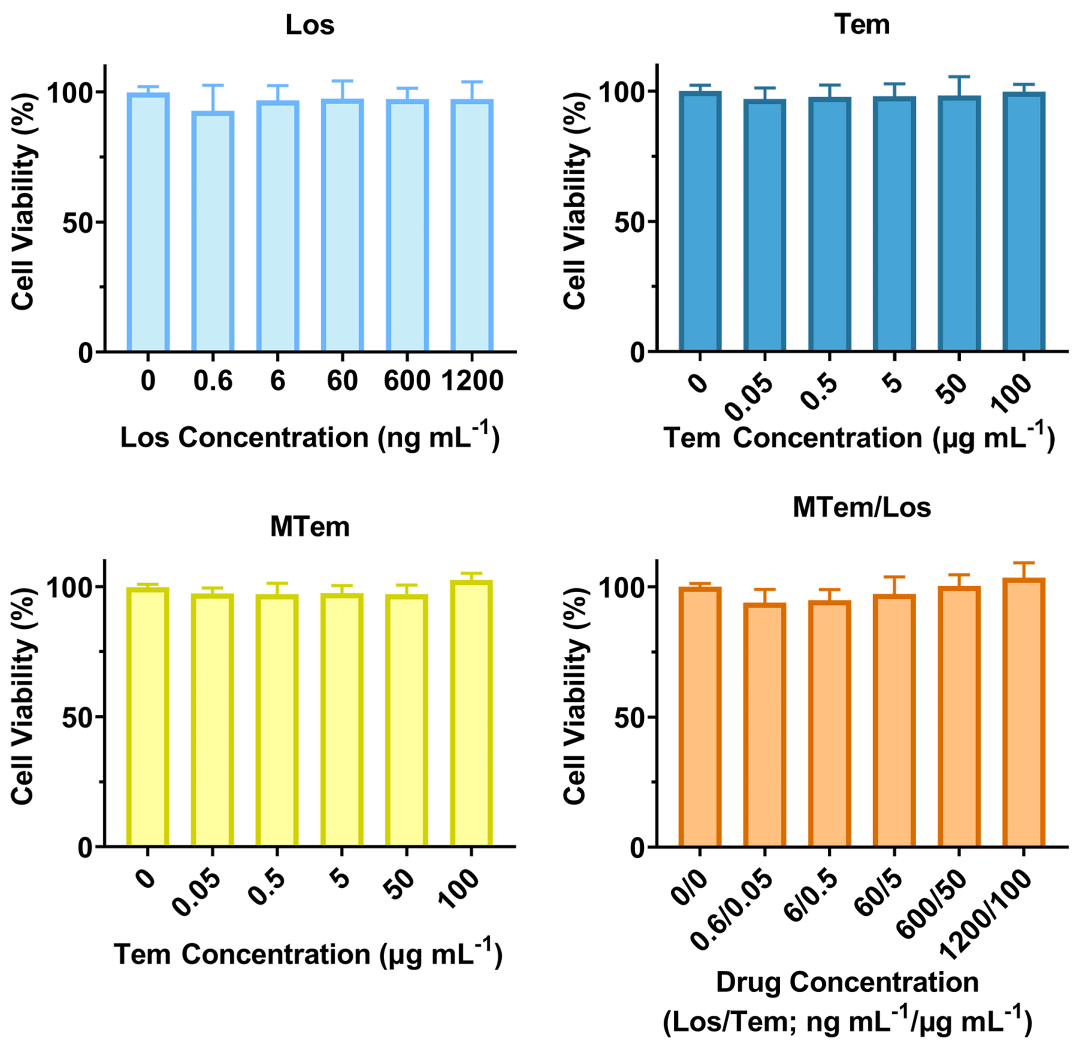

Figure S19. Effect of Los, Tem, MTem, and MTem/Los in the cultured RAW264.7 cells viability by CCK-8 assay. RAW264.7 cells were treated with Los, Tem, MTem, and MTem/Los (with a final concentration of Los/Tem: 0 ng mL^{-1}/0 μg mL^{-1}, 0.6 ng mL^{-1}/0.05 μg mL^{-1}, 6 ng mL^{-1}/0.5 μg mL^{-1}, 60 ng mL^{-1}/5 μg mL^{-1}, 600 ng mL^{-1}/50 μg mL^{-1}, and 1200 ng mL^{-1}/100 μg mL^{-1}) for 24 h, followed by CCK-8 assay. Data were presented as mean ± SD; n = 5.

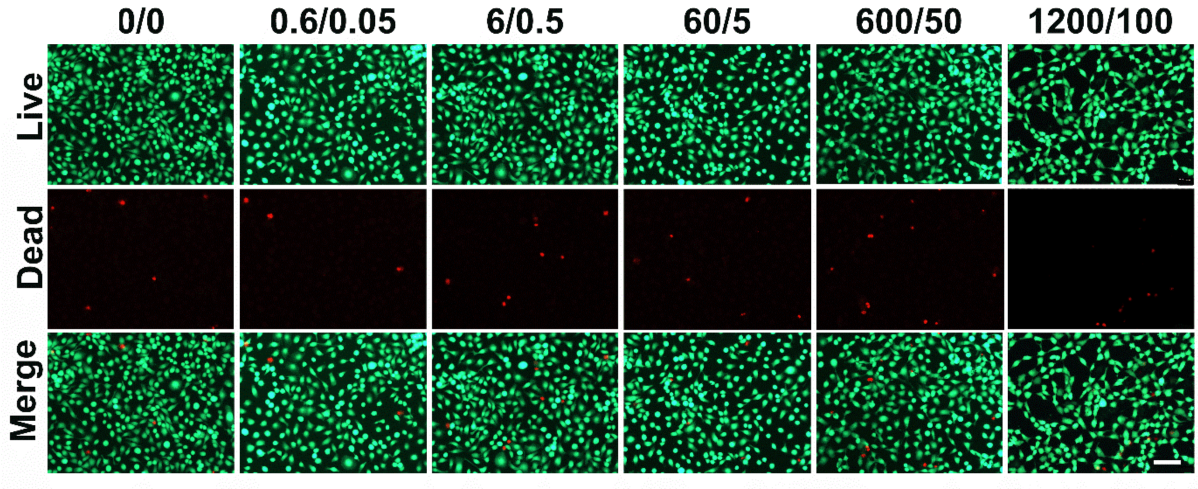

Figure S20. Representative Live/Dead images of HCECs after 24 h treatment of different concentrations of MTem/Los (with a final concentration of Los/Tem: 0 ng mL^{-1}/0 μg mL^{-1}, 0.6 ng mL^{-1}/0.05 μg mL^{-1}, 6 ng mL^{-1}/0.5 μg mL^{-1}, 60 ng mL^{-1}/5 μg mL^{-1}, 600 ng mL^{-1}/50 μg mL^{-1}, and 1200 ng mL^{-1}/100 μg mL^{-1}). Green: live cells; Red: dead cells. The scale bar is 100 μm.

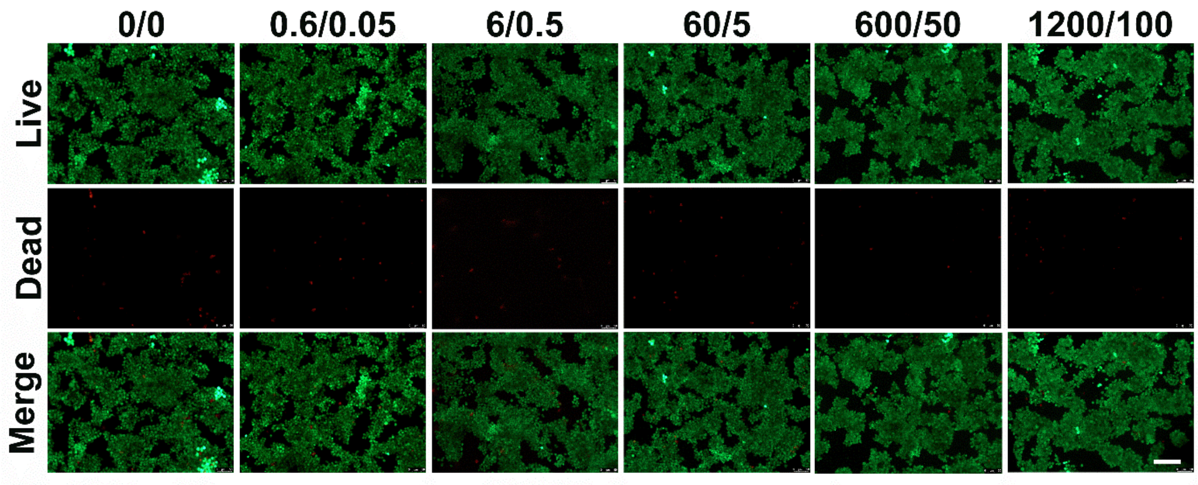

Figure S21. Representative Live/Dead images of RAW264.7 cells after 24 h treatment of different concentrations of MTem/Los (with a final concentration of Los/Tem: 0 ng mL^{-1}/0 μg mL^{-1}, 0.6 ng mL^{-1}/0.05 μg mL^{-1}, 6 ng mL^{-1}/0.5 μg mL^{-1}, 60 ng mL^{-1}/5 μg mL^{-1}, 600 ng mL^{-1}/50 μg mL^{-1}, and 1200 ng mL^{-1}/100 μg mL^{-1}). Green: live cells; Red: dead cells. The scale bar is 100 μm.

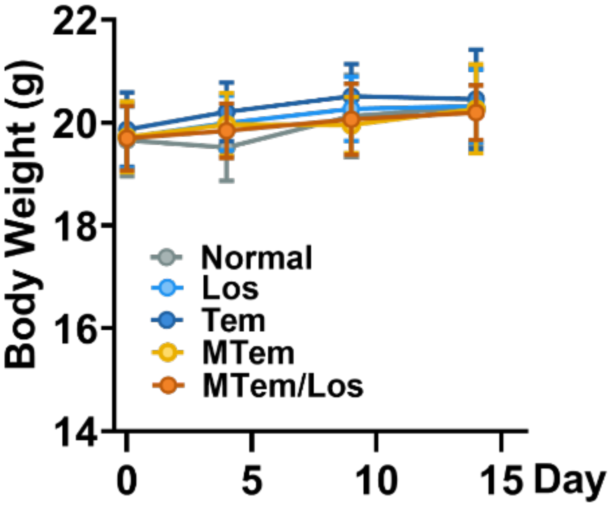

Figure S22. Daily records of mice body weights in Normal, Los, Tem, MTem, and MTem/Los groups at 0, 4, 9, and 14 days of administration throughout the 2-week safety evaluation process. Data were presented as mean ± SD; n = 10.

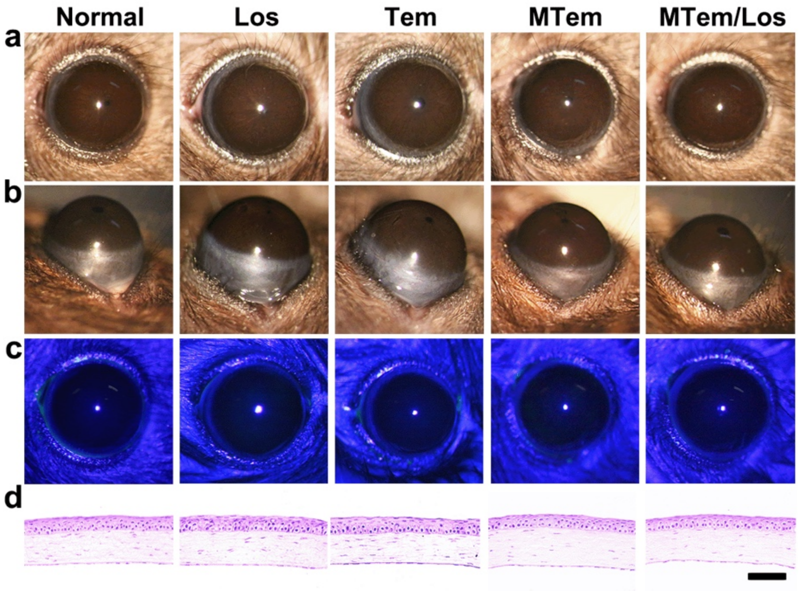

Figure S23. *In vivo* biosafety of 0.9% saline (w/v), Los (15.6 μg kg^{-1}), Tem (2.5 mg kg^{-1}), MTem (Tem: 2.5 mg kg^{-1}), MTem/Los (Tem: 2.5 mg kg^{-1}, Los: 15.6 μg kg^{-1}) to the cornea of mice. Observation of cornea and limbus of bright light (a,b) and cobalt blue light (c) under slit lamp in Normal, Los, Tem, MTem, and MTem/Los group after 14 consecutive days of administration. (d) H&E staining of the cornea from eyes in Normal, Los, Tem, MTem, and MTem/Los groups after 14 days of administration. The scale bar is 100 μm.

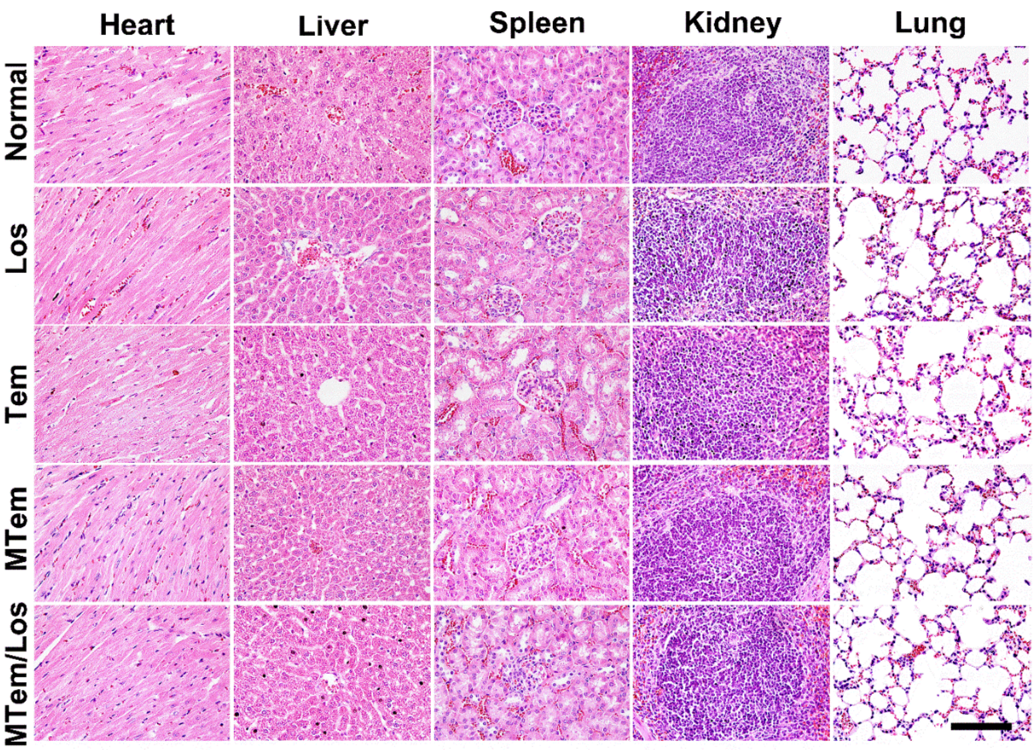

Figure S24. H&E staining of histological sections of heart, liver, spleen, kidney, and lung samples collected on the 30th day after diverse treatments including 10 μL of saline (w/v), Los (15.6 μg kg^{-1}), Tem (2.5 mg kg^{-1}), MTem (Tem: 2.5 mg kg^{-1}), MTem/Los (Tem: 2.5 mg kg^{-1}, Los: 15.6 μg kg^{-1}) onto the eye surface twice per day. The scale bar is 100 μm.
